# Inhibition of the Autophagy Pathway Synergistically Potentiates the Cytotoxic Activity of Givinostat (ITF2357) on Human Glioblastoma Cancer Stem Cells

**DOI:** 10.3389/fnmol.2016.00107

**Published:** 2016-10-27

**Authors:** Francesca Angeletti, Gianluca Fossati, Alessandra Pattarozzi, Roberto Würth, Agnese Solari, Antonio Daga, Irene Masiello, Federica Barbieri, Tullio Florio, Sergio Comincini

**Affiliations:** ^1^Department of Biology and Biotechnology, University of PaviaPavia, Italy; ^2^Preclinical Research Department Italfarmaco Research Center, Italfarmaco S.p.ACinisello Balsamo, Italy; ^3^Department of Internal Medicine, Centre of Excellence for Biomedical Research, University of GenovaGenova, Italy; ^4^Regenerative Medicine, IRCCS Azienda Ospedaliera Universitaria San Martino - ISTGenova, Italy

**Keywords:** glioblastoma multiforme, programmed cell death, histone deacetylase inhibitor, cancer stem cell, autophagy

## Abstract

Increasing evidence highlighted the role of cancer stem cells (CSCs) in the development of tumor resistance to therapy, particularly in glioblastoma (GBM). Therefore, the development of new therapies, specifically directed against GBM CSCs, constitutes an important research avenue. Considering the extended range of cancer-related pathways modulated by histone acetylation/deacetylation processes, we studied the anti-proliferative and pro-apoptotic efficacy of givinostat (GVS), a pan-histone deacetylase inhibitor, on cell cultures enriched in CSCs, isolated from nine human GBMs. We report that GVS induced a significant reduction of viability and self-renewal ability in all GBM CSC cultures; conversely, GVS exposure did not cause a significant cytotoxic activity toward differentiated GBM cells and normal mesenchymal human stem cells. Analyzing the cellular and molecular mechanisms involved, we demonstrated that GVS affected CSC viability through the activation of programmed cell death pathways. In particular, a marked stimulation of macroautophagy was observed after GVS treatment. To understand the functional link between GVS treatment and autophagy activation, different genetic and pharmacological interfering strategies were used. We show that the up-regulation of the autophagy process, obtained by deprivation of growth factors, induced a reduction of CSC sensitivity to GVS, while the pharmacological inhibition of the autophagy pathway and the silencing of the key autophagy gene *ATG7*, increased the cell death rate induced by GVS. Altogether these findings suggest that autophagy represents a pro-survival mechanism activated by GBM CSCs to counteract the efficacy of the anti-proliferative activity of GVS. In conclusion, we demonstrate that GVS is a novel pharmacological tool able to target GBM CSC viability and its efficacy can be enhanced by autophagy inhibitory strategies.

## Introduction

Glioblastoma (GBM) is fatal, highly invasive brain tumor still displaying poor prognosis (Ohgaki and Kleihues, 2013) even after aggressive multi-modal therapy, including neurosurgery, radiotherapy, and chemotherapy with temozolomide (Stupp et al., 2005). Different GBM features are responsible for the therapeutic failure: the peculiar structure of the brain and the invasive behavior of GBM prevent complete surgical tumor excision; the brain blood barrier prevents systemically administered chemotherapeutics to reach the central nervous system (CNS) in clinically effective concentrations (Omuro and Deangelis, 2013), the molecular complexity of GBM showing a variety of genetic alterations, strongly influencing the therapy outcome, since different mutations might determine different drug sensitivity (Brennan et al., 2013; Patel et al., 2014). In addition, the presence of cancer stem cells (CSCs) within the tumor mass is responsible for the recurrence after therapy (Vescovi et al., 2006; Florio and Barbieri, 2012). CSC theory proposes that tumor development is dependent on a small cell population endowed with self-renewal and multilineage differentiation ability, a strong resistance to conventional chemotherapy, and the ability to propagate the tumor when xenografted in animal models (Wurth et al., 2014). Since the most aggressive or refractory cancers contain the highest number of CSCs, the therapeutic importance of the eradication of this particular cancer cell subpopulation is a relevant research goal to overcome GBM therapy resistance (Al-Hajj et al., 2004; Singh et al., 2004).

The wide variety of genetic alterations in GBM necessitates the use of strategies targeting processes like chromatin remodeling, which can revert the altered status of multiple genes. The wide range of cellular and molecular effects mediated by histone deacetylase inhibitors (HDACi) make these drugs suitable candidates for the treatment of such heterogeneous tumors (Lee et al., 2015). HDACi act through the inhibition of histone deacetylases, enzymes that control chromatin remodeling and acetylation of histone and non-histone proteins (Ververis and Karagiannis, 2012). Pharmacologically-induced unbalanced acetylation (histone acetyl transferase activity) and deacetylation (HDAC activity) in favor of the former, creates an hyperacetylated status, promotes a relaxed chromatin structure and an accessible DNA backbone for the transcriptional machinery favoring the activation/repression of gene transcription (Peart et al., 2005), by which HDACi induce cell death (Bolden et al., 2013), cell cycle arrest, senescence (Pazolli et al., 2012), differentiation, or autophagy (Robert et al., 2011) in tumor cells. In GBM cells, HDACi reduce proliferation *via* cell cycle arrest and apoptosis, suppress tumor growth in experimental *in vivo* models, and potentiate the effects of radiotherapy, cytotoxic agents and immune-therapeutics (Thurn et al., 2011). Several HDACi, including SAHA, trichostatin A, valproic acid, belinostat, have been tested in GBM models, and several clinical trials, based on HDACi monotherapy or as drug association strategies are concluded or ongoing (De Souza and Chatterji, 2015).

We report the efficacy of givinostat (GVS), a pan-histone deacetylase inhibitor, on human GBM CSC viability and self-renewal and the involvement of apoptosis and macroautophagy (hereafter referred as autophagy) in this response.

## Materials and methods

### Tumor samples, cell cultures, and chemicals

Nine glioma post-surgical specimens were obtained from the Neurosurgery Department of the IRCCS-AOU San Martino IST, (Genova, Italy) after patients' informed consent and Institutional Ethical Committee approval. All patients underwent surgery for the first time and never received chemo- or radio-therapy. Tumors were derived from 6 males and 3 females and the mean age was 57.5 years. Pathological analysis classified gliomas as grade IV glioblastoma (*n* = 8), or grade III anaplastic astrocytoma (*n* = 1) according to World Health Organization criteria. Cell cultures deriving for each tumor sample were coded as GBM1 to GBM9. Patients and tumors details are reported in Supplementary Table 1.

All GBM-derived CSCs were previously isolated and characterized (Gatti et al., 2013; Wurth et al., 2013). Tumor samples were immediately processed to obtain cell cultures enriched in CSCs. Briefly, cell suspension obtained after mechanical dissociation, was filtered through a 40 μm strainer (BD Biosciences, San Jose, CA, USA) to remove aggregates, and cultivated in serum-free medium containing DMEM-F12/Neurobasal (1:1), B27 supplement (Gibco-Thermofisher, Paysley, UK), 2 mM L-glutamine (Lonza, Basel, Switzerland), 1% penicillin-streptomycin (Lonza), 15 μg/ml insulin (Sigma-Aldrich, St.Louis, MO, USA), 2 μg/ml heparin (Sigma-Aldrich) and completed with recombinant human bFGF (10 ng/ml; Miltenyi Biotec, Cologne, Germany) and EGF (20 ng/ml; Miltenyi Biotec) (Bajetto et al., 2013). This medium is defined as “complete medium.” These cells gave rise to floating tumor-spheres after 2 weeks, but can also growing as stem cells in monolayer, after spheres disaggregation and in presence of Matrigel (BD Biosciences, San Jose, CA, USA), without losing expression of stem cell markers, spherogenic properties, differentiation and tumorigenic potential (Griffero et al., 2009).

All the cell cultures analyzed in this study were previously characterized for tumor-initiating capacity by orthotopic xenograft, induced by injection of 10,000 sphere-derived cells in 6–8-weeks old non-obese diabetic severe combined immunodeficient (NOD/SCID) mice (Charles River Laboratories, Wilminglon, MA, USA), as detailed in previous studies (Carra et al., 2013; Gritti et al., 2014; Corsaro et al., 2016). Animals were housed in pathogenic-free conditions, and handled in agreement with the institutional and national guidelines for the care and use of laboratory animals (Italian D.lgs 26/2014); the experimental plan was approved by the IRCCS AOU S. Martino-IST (Genova, Italy) Institutional Animal Care and Use Committee (IACUC).

To induce differentiation, GBM CSC cultures were seeded and maintained for 2 weeks in DMEM/F12 supplemented with 2 mM L-glutamine, penicillin-streptomycin and 10% FBS (Euroclone, Milano, Italy). Deprivation of growth factors was induced removing bFGF, EGF, and the B27 supplement from the culture medium.

Three human GBM established cell lines were also used: T98G, U373-MG, and U138-MG (ATCC). GBM cell lines were grown in DMEM supplemented with 2 mM L-glutamine, penicillin-streptomycin and 10% FBS.

Human umbilical cords (*n* = 2) were collected from full-term women, immediately after cesarean section at the Gynecology Department of International Evangelical Hospital (Genova, Italy), after informed consent and approval by Institutional Ethic Committee. After vessel removal, cords were digested with collagenase I-S (0.5 μg/ml, Sigma-Aldrich) for 1 h to expose Wharton-Jelly and isolated cells cultured in DMEM (10% FBS, 2 mM L-Glutamine). MSCs were used after full characterization by flow cytometry (MSC Phenotyping Kit, Miltenyi Biotec), as defined by International Society for Cellular Therapy (Dominici et al., 2006).

The pan-HDAC inhibitor givinostat (ITF2357) was kindly provided by Italfarmaco S.p.A. (Cinisello Balsamo, Italy). Givinostat was dissolved in DMSO at the stock concentration of 10 mM and for all experiments it was diluted in the specific medium to obtain the final concentrations. Rapamycin (Cell Signaling Technology, Danvers, MA, USA) and bafilomycin-A1 (Sigma-Aldrich), were used to promote the induction and inhibition of autophagy, respectively. Both were dissolved in DMSO at the stock concentration of 100 μM, and for all experiments drugs were diluted in the specific medium to obtain the appropriate concentration. Controls received the same amount of residual DMSO (not exceeding 0.1%) than treated samples.

### Sphere-formation assay

GBM CSCs were seeded in complete medium without Matrigel, in 48-well plates at 1000 cells/well. After 24 h cells were exposed to increasing concentration of GVS (0.1–2 μM), and sphere-formation capacity was monitored after 7 days, to allow spheres generation. The number of spheres in wells was quantified using a digital camera mounted on a transmitted light microscope and visually calculated by three independent operators. To further demonstrate the inhibitory activity of GVS on sphere-formation process, GBM-derived tumor-spheres, generated in the absence or presence of GVS (0.1–0.5 μM), were disaggregated and re-plated in fresh medium avoid of GVS. Spheres-formation efficiency (SFE) (Wurth et al., 2016), calculated as the number of formed spheres/1000 plated cells, was re-evaluated after 7 days.

### MTT and trypan-blue dye exclusion assays

Mitochondrial activity, as index of cell viability, was evaluated by measuring the reduction of 3-(4,5-dimethylthiazol-2-yl)-2,5-diphenyltetrazolium bromide (MTT, Sigma-Aldrich,). GBM CSCs were plated into 96 or 48-well plates (pre-coated with Matrigel), and the number of cells/well (ranging from 1000 to 5000) was adjusted depending on different proliferation rates and time of treatment. At the end of each treatment, cells were incubated with MTT solution (2.5 mg/ml), for 2 h. After MTT removal, the formed purple formazan crystals were dissolved in DMSO, and absorbance measured at 570 nm wavelength (Pattarozzi et al., 2008).

Trypan blue exclusion assay was used to evaluate cell viability reduction induced by GVS treatment. Initially, 5000 cells/well were seeded in 24-well plates, pre-coated with Matrigel. After 24 h, cells were treated with increasing concentration of GVS for additionally 48, 72, and 96 h. Every 24 h, viable cells were counted in the presence of Trypan blue 0.4% w/v (Bio-Rad, Marnesla-Coquette, France), to distinguish between dead and live cells, using the TC-20® automated cell counter (Bio-Rad) (Villa et al., 2016).

### Drug synergism evaluation

To determine potential synergistic drug effects on growth of CSCs GBM1, GBM2, and GBM3, cells were exposed to various combinations of GVS (0.5 μM) and bafilomycin-A1 (1.25–50 nM). Cell viability was determined by MTT assay after 48 and 72 h of treatment. Drug interactions (synergistic, additive, antagonistic) were determined by the median effect analysis method, as described (Chou and Talalay, 1984; Chou, 2010), using the CompuSyn software (ComboSyn Inc., Paramus, NJ, USA). This approach takes into account the potency, the shape, and the slope of the dose-dependent neutralization curve of each drug alone and in combination, to calculate a combination index (CI). A CI value of 1 indicates an additive effect, <1 indicates synergism, and >1 indicates antagonism.

### Annexin V/PI double staining

GBM CSCs were treated with GVS and bafilomycin-A1 alone or in association for 48 or 72 h, depending on the experiments. After the treatment, cells were washed gently with PBS, trypsinized, pelleted, secondly washed in PBS and finally counted. According to Annexin V-FITC Apoptosis detection Kit (eBioscience, Hatfield, UK), cells were resuspended in the Annexin Binding Buffer at the concentration of 2–5 × 10^5^ cells/ml, then 5 μl of Annexin-V was added to 195 μL of the cell suspension and incubated for 15 min, at the room temperature and protected from direct light. Propidium Iodide (20 μg/ml) was added and cells were analyzed by flow cytometry (FACSCanto II flow cytometer, BD Biosciences) recording 10,000 events per sample. Annexin V-positive cells were considered in the early stages of apoptosis, whereas cells in the late stages of apoptosis were Annexin V- and PI-positive (Carra et al., 2013).

### Real-time PCR expression analysis

Total RNA from GBM CSCs was extracted using the Aurum Total RNA Mini Kit (Bio-Rad), according to the manufacturer's instruction, and reverse transcribed into cDNA using the iScript cDNA Synthesis Kit (Bio-Rad). Single stranded cDNA products were analyzed by Real-time PCR using the SsoFast^TM^ Eva Green Supermix (Bio-Rad,) on a CFX96 Touch Real-time PCR (Bio-Rad). Cycling conditions were set at 94°C for 30 s, 60°C for 30 s and 72°C for 30 s, for 37 cycles. Primers, for *MAP1LC3B* amplification, were pre-designed by PrimePCR (Bio-Rad). Human *HPRT1* and *TBP* pre-designed primers (Bio-Rad) were used as internal controls. Levels of target genes in each sample were normalized on the basis of *HPRT1* and *TBP* amplification and reported as relative values (Mathur, 2014).

### Immunoblotting analysis

Following specific treatments, cells were lysed in buffer containing 1% Igepal, 20 mM Tris–HCl, pH 8, 137 mM NaCl, 10% glycerol, 2 mM EDTA, 1 mM phenylmethylsulfonyl fluoride, 1 mM sodium orthovanadate, 10 mM NaF (all from Sigma-Aldrich), and the “Cømplete protease inhibitor mixture” (Roche) for 20 min at 4°C. Nuclei were removed through centrifugation (5000 rpm at 4°C for 10 min); total protein concentration was measured with Bradford assay (Bio-Rad). Proteins (40–60 μg) were resuspended in Laemmli buffer (2% SDS, 62.5 mM Tris, pH 6.8, 0.01% bromophenol blue, 1.43 mM β2-mercaptoethanol,and 0.1% glycerol) and were separated on 10 or 12% (depending on protein size) SDS-PAGE, and subsequently transferred onto PVDF membrane (Bio-Rad) (Massa et al., 2004). The following antibodies were used: anti-LC3B, anti-Beclin1, anti-Atg7, and anti-acetyl-α-tubulin (Lys40) (all from Cell Signaling Technologies). β-actin (Cell Signaling Technologies) and α-tubulin (Sigma Aldrich) were used as internal control to ensure equal loading and transfer of proteins. Antibodies were all diluted at 1:1000 in 3% bovine serum albumin (BSA), except α-tubulin which was diluted 1:7500 in Tween 20 (0.1%)/PBS. Species-specific peroxidase-linked ECL secondary antibodies (GE Healthcare USA, 1:5000 dilutions) were used. Protein signals and densitometric analysis were performed using the Clarity Western ECL substrate (BioRad) and the Chemi-Doc System (Bio-Rad).

### LC3B-GFP autophagosome and electron microscopy analysis

For autophagosome detection, GBM CSCs were seeded at the density of 5000 cells/well in 48-well plates pre-coated with Matrigel. After 12 h, cells were transduced with BacMam LC3B-GFP viral particles (multiplicity of infection, MOI = 30), according to the Premo Autophagy Sensor Kit (Invitrogen, Carlsbad, CA, USA). After 16 h of incubation, cells were treated with GVS (0.5 μM), bafilomycin-A1 (10 nM, added 4 h before the end of the time interval), the combination of the two drugs, or vehicle (controls); LC3B-GFP signals were monitored after additional 24 h, using an inverted fluorescence microscope (40X magnification, Eclipse Nikon TS100, Minato, Japan). GFP-positive vesicles were analyzed in number and shape (area) using the Autocounter tool as described (Fassina et al., 2012).

For ultrastructural analysis, GBM CSCs were grown at 75% confluence, and treated with GVS (0.5 μM) or vehicle. Samples were prepared according to Marchesi et al. (2014). Specifically, after 48 h p.t, cells were harvested by centrifugation at 800 rpm for 3 min and fixed with 2% glutaraldehyde in medium, maintained for 2 h at room temperature. Cells were then rinsed in PBS (pH 7.2) overnight and post-fixed in 1% aqueous OsO_4_ for 2 h at room temperature. Cells were pre-embedded in 2% agarose in water, dehydrated in acetone, and finally embedded in epoxy resin (Electron Microscopy Sciences, EM-bed812). Ultrathin sections (50–60 nm) were collected on formvar-carbon-coated nickel grids and stained with uranyl acetate and lead citrate. The specimens were finally observed with a Zeiss EM900 electron microscope equipped with a 30 μm objective aperture and operating at 80 kV.

### Cell transfection and Atg7 silencing

To modulate Atg7 protein expression, GBM2 CSCs (80% confluence) were transiently transfected with Lipofectamine LTX reagent (Invitrogen) in presence of pooled Silencer select validated si*ATG7* sequences (s20650, s20651; Ambion, USA) at the final concentration of 150 nM or with the mock solution alone, as negative control. To perform the viability assay (MTT), cells were plated in 96-well plates (2000 cells/well) and assayed after 24, 48, and 72 h p.t. For protein lysates, transfection was performed on cells seeded on 60 mm petri dishes with the same concentration of pooled si*ATG7* sequences.

### Statistical analysis

All reported experiments were carried out in triplicate and performed at least three times. Data were reported as means ± SEM. All statistical analysis (ANOVA followed by Dunnett's *post-hoc* test, or unpaired two-tailed Student's *t*-test), were calculated with Graph-Pad Prism 5.0. *p* ≤ 0.05 was considered statistically significant.

## Results

### GVS affects the viability of GBM cell lines and CSC enriched cultures

Different established human GBM cell lines (i.e., U87-MG, U138-MG, and T98G) were initially tested to evaluate the effect of GVS on cell viability, using the MTT assay. Time-course (from 24 to 96 h) and dose-response (GVS 0.1–2 μM) experiments were performed. GVS reduced cell viability in a concentration- and time-dependent manner (Supplementary Figure 1), although no effects were observed for short time treatments (24 h) or GVS concentrations lower than 0.25 μM.

**Figure 1:**
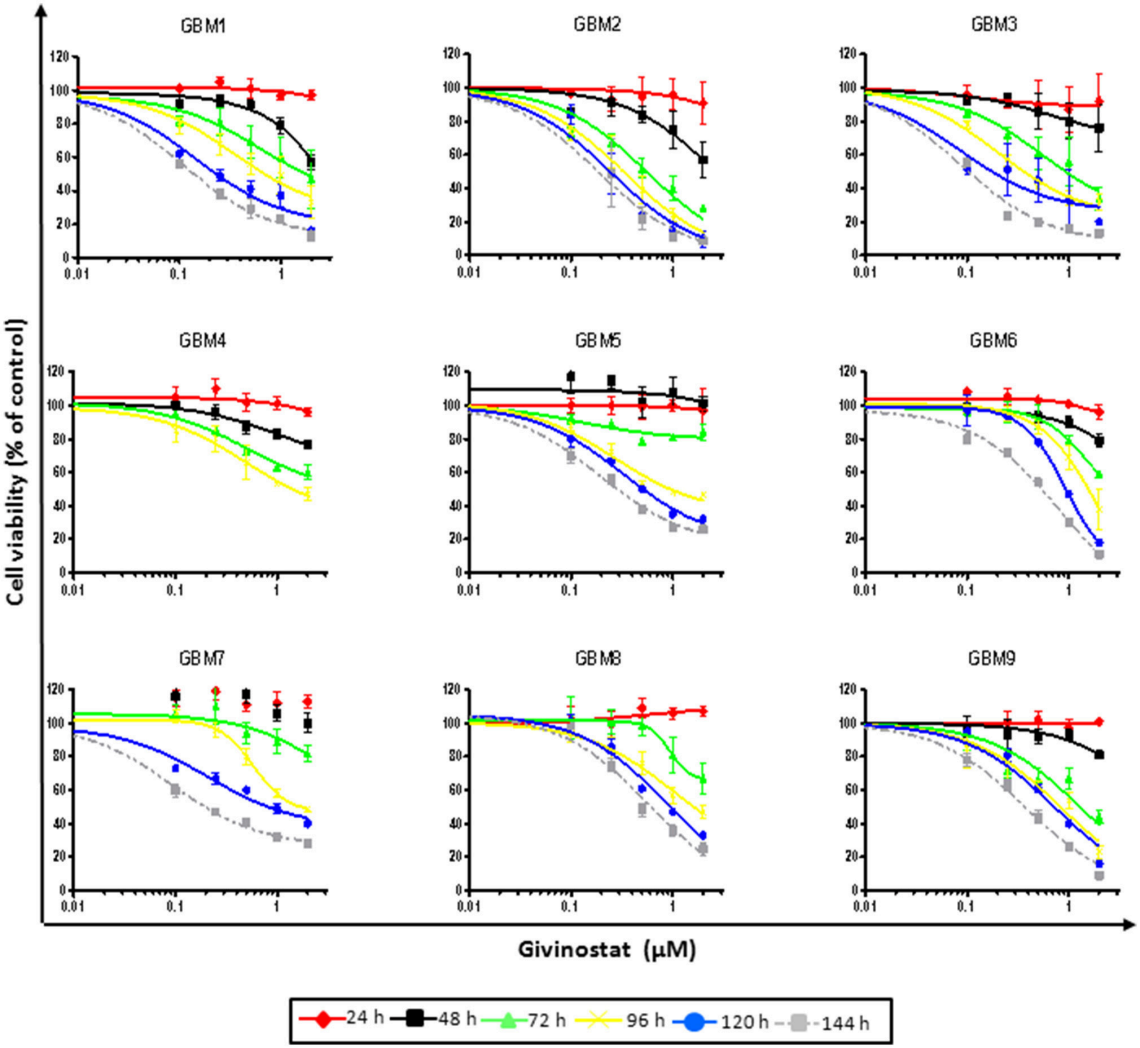
**GVS dose-response curves (0.1–2.0 μM) in nine adherent GBM cell cultures enriched in CSCs**. Cell viability was determined by MTT assay after 24–144 h of treatment. Experiments were performed in triplicate and percentage of inhibition was calculated vs. untreated control cells.

Due to the higher translational potential of GBM CSCs compared to cell lines (Lee et al., 2006), we confirmed the effect of GVS on the viability of CSC-enriched cultures isolated from nine human GBMs. GVS induced anti-proliferative effects in all CSC cultures grown as monolayers in a concentration- and time-dependent manner, with, also in this case, lower efficacy observed for shorter treatments (Figure 1, Supplementary Table 2). The mean GVS IC_50_, calculated after 72 h of treatment, was 0.85 μM but this value decreased proportionally with GVS exposure time, confirming the time-dependent activity of the drug (Table 1).

**Table 1:** **IC_50_ value (microM) of GBM CSCs exposed for different time intervals to GVS**.

**CSCs**	**72 h**	**96 h**	**120 h**	**144 h**
GBM1	0.63	0.35	0.15	0.11
GBM2	0.55	0.33	0.24	0.19
GBM3	0.56	0.20	0.09	0.09
GBM4	0.51	0.49	–	–
GBM5	Not reached	0.28	0.32	0.19
GBM6	1.47	0.93	0.93	0.66
GBM7	1.19	1.54	0.20	0.09
GBM8	1.23	1.01	0.89	0.57
GBM9	1.29	0.83	0.72	0.37
MEAN IC_50_	0.85	0.66	0.44	0.30
VALUE (RANGE)	(0.51–1.47)	(0.20–1.54)	(0.09–0.93)	(0.09–0.66)

Calculations were performed using GraphPad Prism software. (Not reached, absence of a significant GVS activity; empty boxes, not performed).

Growing CSCs as monolayer on Matrigel allows for better cellular and biochemical characterization without interfering with stem-like characteristics (Griffero et al., 2009; Wurth et al., 2013), although the ability to grow as tumor-spheres is a defining feature of these cells. Thus, we verified whether this experimental condition interfere with CSC drug responsivity. Comparing GVS (0.1–2 μM) effects on CSCs grown as either tumor-spheres or monolayer, we observed no differences in drug sensitivity (data not shown).

The cellular and molecular mechanisms of GVS activity on GBM CSCs were analyzed in the most responsive CSC cultures (GBM1, GBM2, and/or GBM3) (Table 1), in comparison, in selected experiments, with less responsive cultures (GBM7, GBM8, GBM9). To evaluate the contribution of cytostatic or cytotoxic effects of GVS, GBM1, and GBM2 CSCs were assayed by Trypan blue exclusion test, after treatment with GVS (0.5–2 μM for 48–96 h). Forty-eight hour treatment caused cytostatic effect at all concentrations tested (with the exception of GBM1 treated with high GVS concentration) with no changes in the number of dead cells compared to untreated cells at T_0_. However, extending treatment time, cells started to die in a concentration- and time-dependent manner (Supplementary Figure 2).

To establish the minimum time of GVS exposure required to decrease CSC viability, we performed cell growth recovery experiments (Favoni et al., 2010). GBM1, GBM2, and GBM3 cultures treated with 0.5 μM GVS, were washed and incubated in drug-free fresh medium. Cell viability by MTT assay determined either immediately after medium replacement or after additional 24–72 h (see Supplementary Figure 3 for the experimental scheme). Twenty-four hour treatment decreased growth rate and the low proliferative activity partly persisted during the following 24 h, but after additional recovery time the cells reacquired an exponential-like growth rate, reaching levels comparable to non-treated cells. The extension of the treatment with GVS to 48 or 72 h delayed the time to recovery causing, after 72 h of treatment, a significant reduction in cell viability (Supplementary Figure 4).

### GVS inhibition of cell survival is specific for CSCs and is diminished after differentiation

GVS anti-proliferative effect was also tested in differentiated GBM cells obtained by shifting CSC cultures from growth factor-enriched to FBS-containing medium. After 14 days, differentiated GBM CSCs lose *in vivo* tumorigenicity and stem cell marker expression and exhibit glial and/or neuronal marker expression (Gritti et al., 2014; Banelli et al., 2015). GVS did not affect the viability of differentiated GBM1 and GBM2 cells (hereafter named GBM1 DIFF and GBM2 DIFF), even after 144 h of treatment with the highest concentration of drug (Supplementary Figure 5A). Furthermore, GVS treatment of two cultures of normal human umbilical cord-derived mesenchymal stem cells (MSCs) caused no reduction of viability at all concentrations and times tested in MSC1, whereas only the highest GVS concentration (2 μM) slightly reduced MSC2 viability (Supplementary Figure 5B). These results confirm that independently from the culturing conditions, the antitumoral effects of GVS are selectively directed against CSC specific proliferation mechanisms, with GBM DIFF cells and MSCs mostly insensitive to the drug.

**Figure 2:**
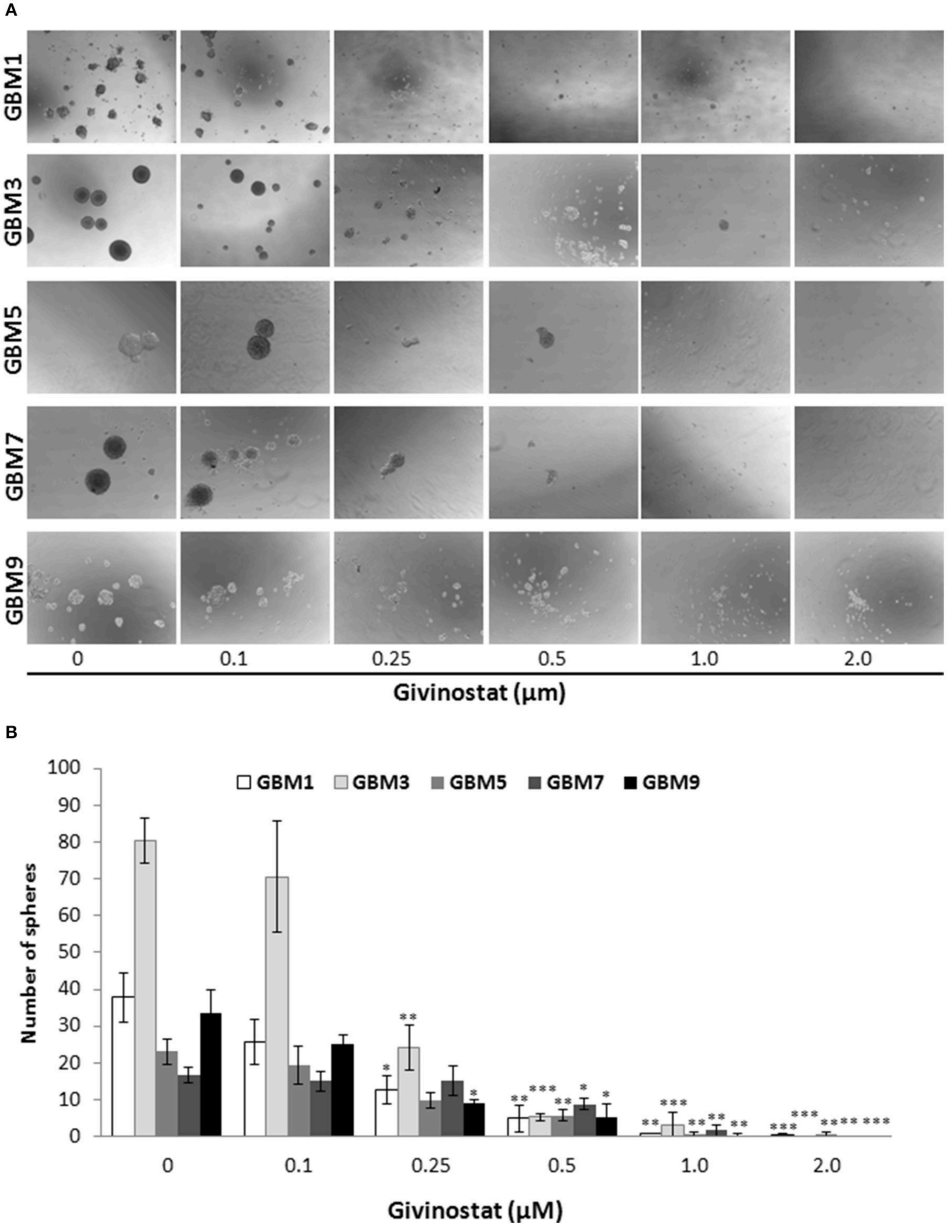
**GVS effect on GBM CSC sphere formation**. **(A)** GBM1, GBM3, GBM5, GBM7, and GBM9 were maintained in the selective medium for stem cells in the absence or presence of increasing concentrations of GVS. Sphere formation was visually monitored and after 14 days the number of spheres/well was counted. Representative microphotographs (magnification 10X) are reported. **(B)** Histogram reports the mean sphere number from four experiments (*p < 0.05, **p < 0.01, ***p < 0.001, ANOVA followed by Dunnett's *post-hoc* test).

**Figure 3:**
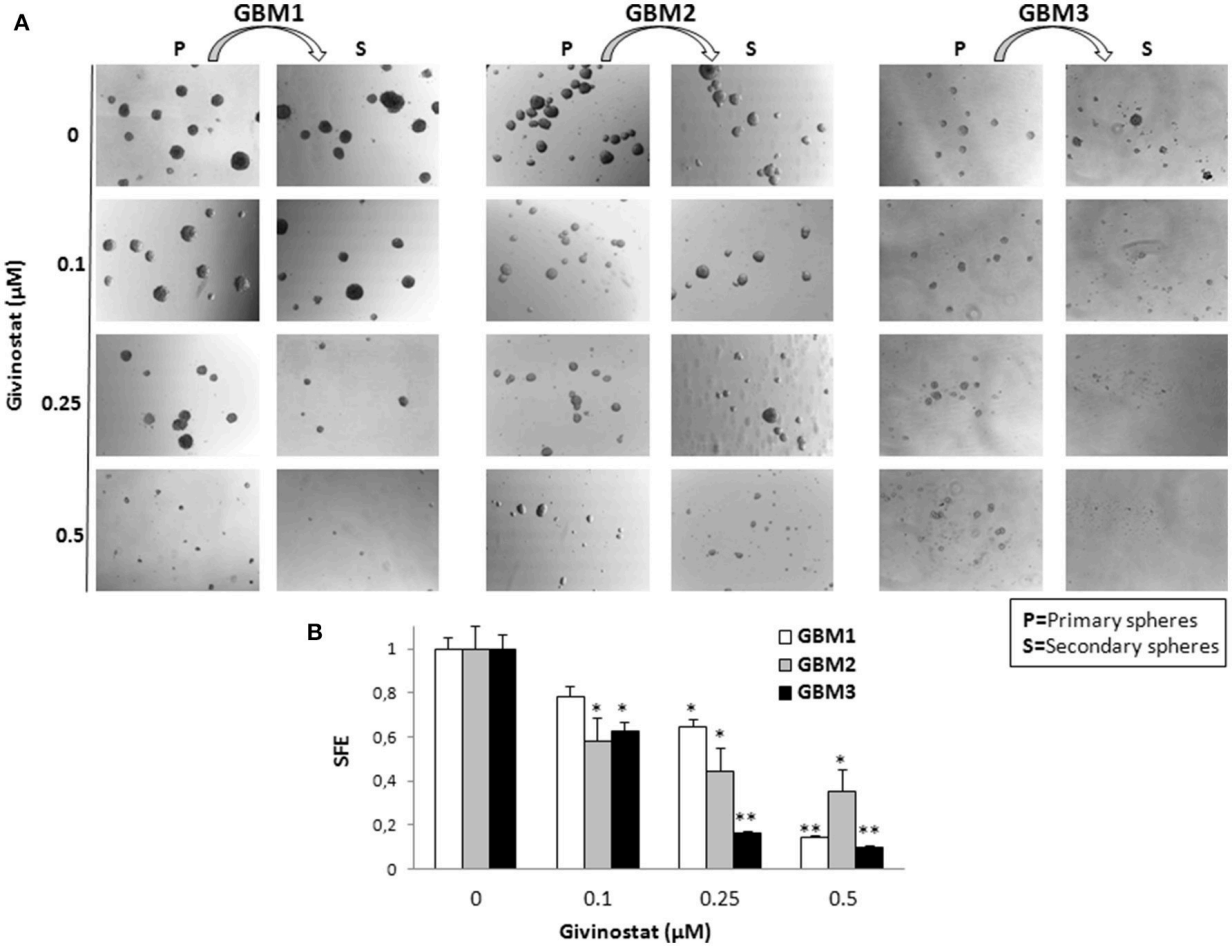
**GVS effect on GBM CSCs on self-renewal measured as secondary sphere formation**. **(A)** GBM1, GBM2, and GBM3 were grown in stem cell permissive conditions for 7 days to allow sphere appearance (*P* = Primary spheres) in the presence or absence of increasing concentration of GVS. Spheres were then disaggregated and single cells were replated in medium without GVS and, following further 7 days, the formation of secondary spheres (S) was evaluated. Microphotographs (magnification 10X) are representative of GVS inhibitory effect on spherogenesis. **(B)** Histograms indicate the sphere-formation efficiency (SFE) of secondary spheres (mean number of spheres/number of cells seeded per well) for each condition against untreated controls. Control value was set at 1 (*p < 0.05 and **p < 0.01 using ANOVA followed by Dunnett's *post-hoc* test).

Self-renewal is a defining feature of both normal and cancer stem cells. Tumor-sphere formation is considered an indirect index of self-renewal of CSCs (Soeda et al., 2008). To understand whether GVS affects GBM CSC self-renewal, we performed a spherogenesis assay. GBM1, GBM3, GBM5, GBM7, and GBM9 CSCs were plated at 1000 cells/well in the presence of increasing concentrations of GVS and allowing to generate spheres for 7 days. As reported in Figure 2A (and quantified in Figure 2B), GVS inhibited spherogenesis in a dose-dependent manner. At low GVS concentrations, tumor-spheres were less compact and organized, while a clear reduction of number and size of spheres was observed on increasing the drug concentration. At the highest concentrations tested (GVS 1 and 2 μM), the inhibition of cell viability in all the cultures was predominant and masked the drug effect on spherogenesis, making difficult the interpretation of the results. A statistically significant reduction of the number of spheres was seen in GBM1, GBM3, and GBM9 starting from the GVS concentration of 0.25–0.5 μM which had low effect on CSC viability, suggesting that GVS inhibition of sphere formation is independent from anti-proliferative effects. An additional experimental approach was performed using GBM1, GBM2, and GBM3 preformed tumor-spheres, which were treated with low GVS concentrations (0.1–0.5 μM) for 7 days, disaggregated and re-plated in GVS-free medium (Figure 3A,B). While untreated CSCs regenerate spheroids, pre-treatment with GVS dose-dependently abolished their spherogenesis ability.

### Effects of GVS on apoptosis/autophagy processes in GBM CSCs

**Figure 4:**
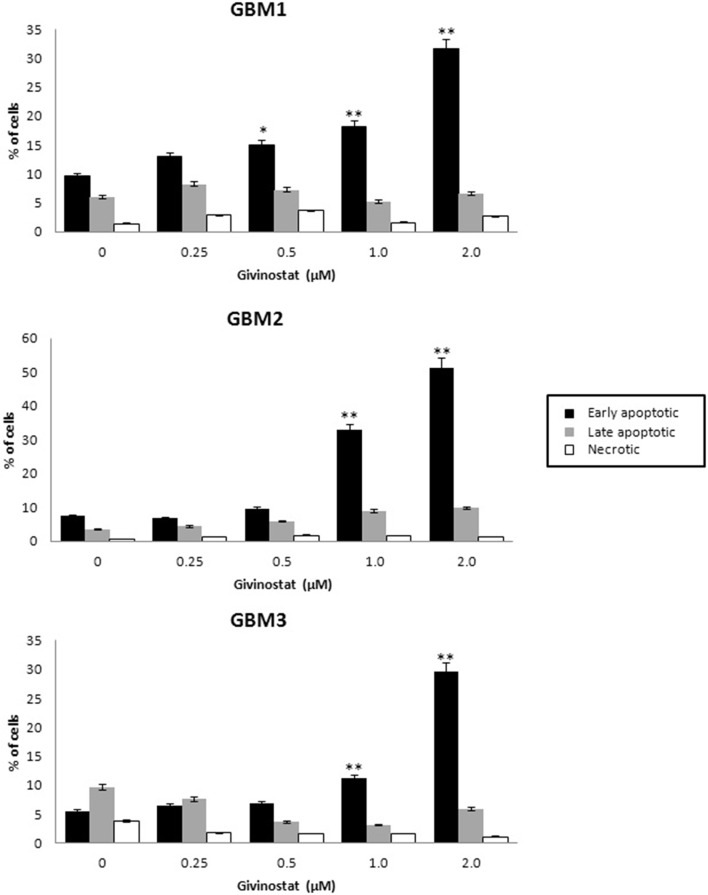
***In vitro* assessment of apoptosis in GBM1, GBM2, and GBM3 CSCs**. FACS analysis of Annexin V-PI staining was used to observe the induction of apoptosis and/or necrosis by GVS. Histograms represent the percentage of each cell population (early apoptotic, late apoptotic and necrotic) after 72 h of GVS treatment (0.25, 0.5, 1.0, and 2.0 μM). Statistical analysis was performed using ANOVA followed by Dunnett's *post-hoc* test (*p < 0.05, **p < 0.01).

GVS was tested for the ability to interfere with apoptosis and autophagy processes. GBM1, GBM2, and GBM3 CSCs, treated with GVS (up to 2 μM) for 72 h, were tested in AnnexinV-PI double staining by FACS analysis (Figure 4), showing a concentration-dependent increase of cells entering the early phases of apoptosis (approximately 30% for GBM1 and GBM3, and 50% for GBM2).

**Figure 5:**
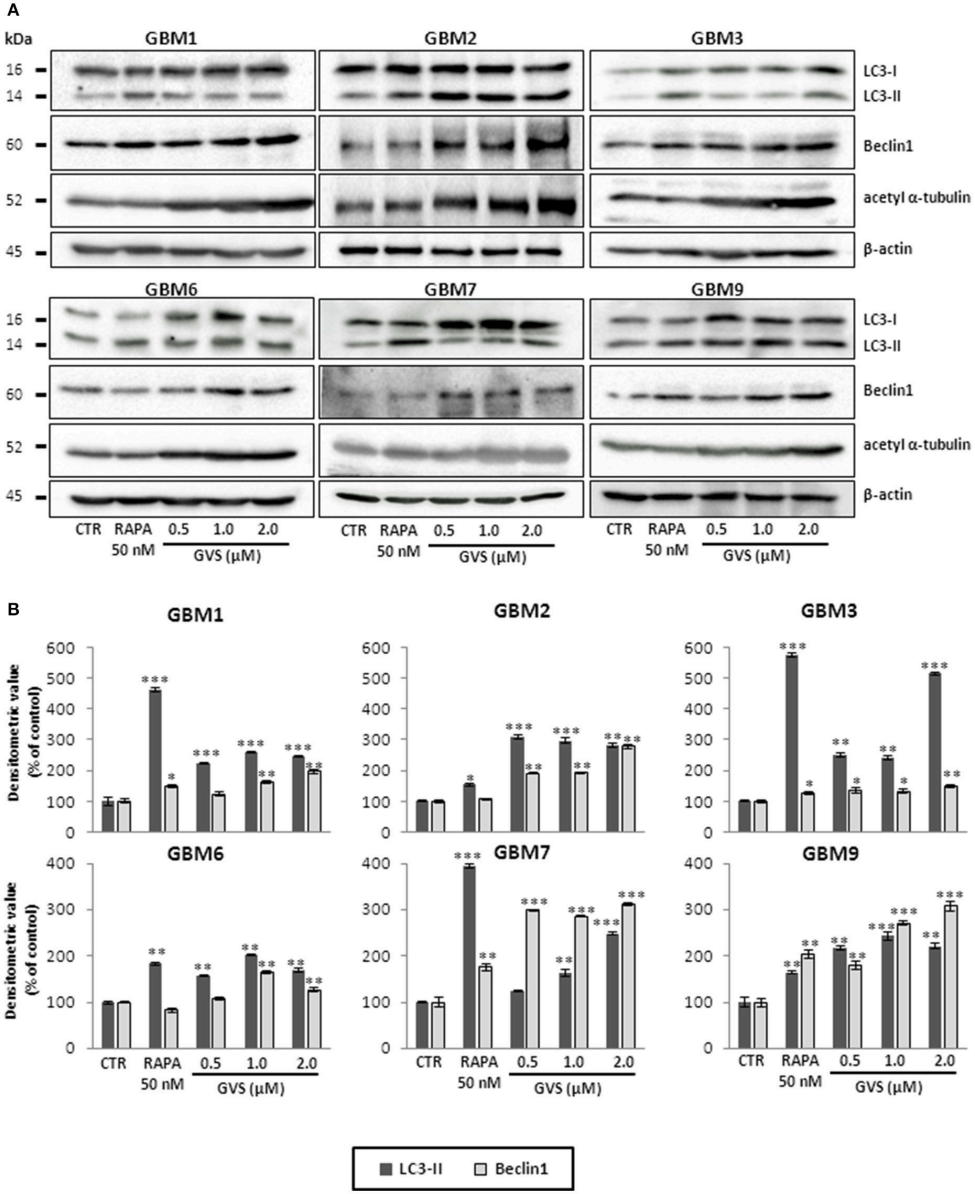
**Modulation of autophagic marker expression following GVS administration in GBM1, GBM2, GBM3, GBM6, GBM7, and GBM9 CSCs**. **(A)** LC3-I, LC3-II, Beclin1 and acetyl-α-tubulin protein levels was determined by immunoblotting. β-actin was used as protein loading control. Lysates were obtained from cells treated for 72 h with GVS (0.5, 1.0, and 2.0 μM) or rapamycin (50 nM). **(B)** Densitometric analysis of protein expression. Values were normalized to β-actin expression and reported as percentage of untreated controls. Experiments were performed in triplicate, data represent mean ± SEM, (*p < 0.05; **p < 0.01, ***p < 0.001, ANOVA followed by Dunnett's *post-hoc* test).

To study GVS modulation of autophagy, as a preliminary evaluation we analyzed the relative expression of *MAP1LC3B*, coding for LC3B, in GVS-treated GBM1, GBM2, and GBM3 CSCs by Real-time PCR. GVS treatment induced a transient increase in *MAP1LC3B* mRNA content (detectable after 24 and 48 h, and back to the baseline after 72 h, Supplementary Figure 6). Then, the effect of GVS on LC3-II and Beclin1 protein content was investigated in GBM1, GBM2, GBM3, GBM6, GBM7, and GBM9 cells. CSCs were treated for 72 h with GVS (0.5–2 μM) or with the autophagy inducer rapamycin (50 nM, for 72 h): LC3-II and Beclin1 showed an increased amount following GVS treatment, often higher than those caused by rapamycin administration (Figure 5). Increased acetyl-α-tubulin content, a direct marker of HDAC6 inhibition (Haggarty et al., 2003), was observed following GVS administration, confirming the efficacy of the drug on its specific target (Figure 5).

**Figure 6:**
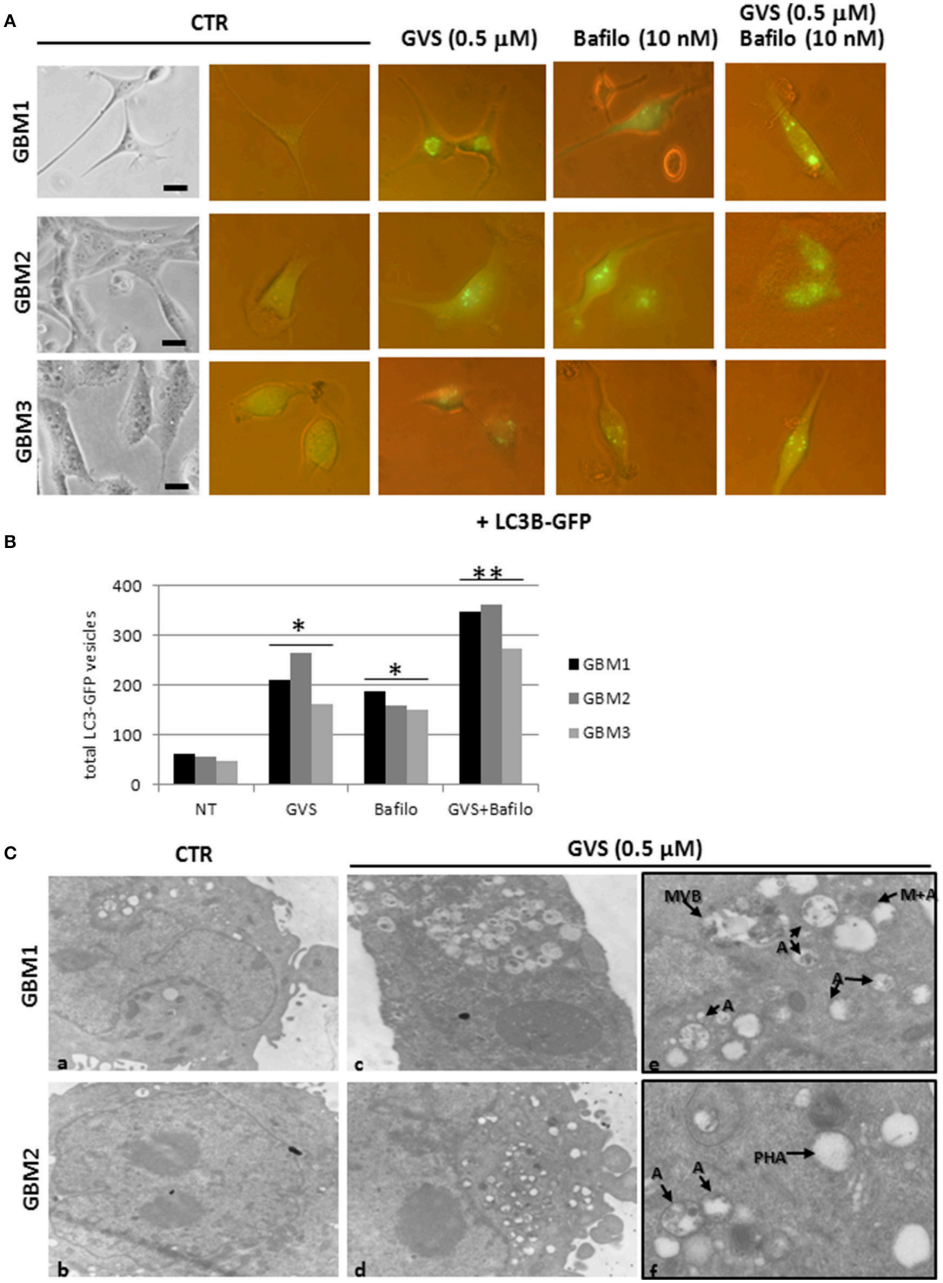
**LC3B-GFP expression in GBM CSCs exposed to GVS**. **(A)** Cells were transduced with baculovirus LC3B-GFP expressing vector. GVS (0.5 μM) was added 16 h after transduction to allow LC3B-GFP expression. Bafilomycin-A1 (10 nM), alone or in combination with GVS (0.5 μM), was added 4 h before the end of the 24 h interval. LC3-GFP signals were monitored using an inverted fluorescence microscope (40X magnification). **(B)** Evaluation of numbers of LC3-GFP vesicles was performed using Autocounter tool. GFP-positive vesicles were considered with area >1 mm^2^ in 50 cells for each treatment. Statistical analysis was performed using ANOVA followed by Dunnett's *post-hoc* test (*p < 0.05, **p < 0.01) comparing GVS, bafilomycin-A1, GVS+bafilomycin-A1 treatments to the respective untreated (CTR) GBM CCSs. **(C)** Representative ultra-structures analysis using transmission electron microphotographs of GBM1 and GBM2 treated with GVS (0.5 μM) for 72 h. GBM1 and GBM2 cells (10^6^) were grown at 75% confluence and treated (**c–f**) or not (**a,b**) with GVS. Cells were harvested, fixed and stained for ultra-structural visualization. Specimens were then observed with a Zeiss EM900 electron microscope. Magnifications are 7000X (**a–d**) and 12000X (**e,f**). “A” indicates large intracellular vesicles, likely autophagosomes; “M+A” indicates a mitochondrion fused with a vesicle (mitophagy), “MVB” indicates a multivesicular body, finally “PHA” indicates a phagophore (isolation membrane) assembly site.

To verify whether GVS-mediated increase in LC3-II and Beclin1 was the resultant of autophagy activation, GBM1, GBM2, and GBM3 CSCs were transduced with a baculovirus LC3B-GFP expressing vector. Cells were treated with GVS (0.5 μM), bafilomycin-A1 (10 nM) or with the combination of the two drugs and analyzed for the amount of GFP-positive vesicles, using the Autocounter tool as described (Fassina et al., 2012). GVS, bafilomycin-A1 and the combined treatment induced significant increases of fluorescent vesicles in the investigated cells; furthermore, the additive results in increasing GFP puncta in the combined GVS+bafilomycin-A1, compared to bafilomycin-A1 alone administration, suggested a direct increase of the autophagy flux induced by GVS (Figures 6A,B). Transmission electron microscope analysis of GVS-treated cells, revealed a high number of double membrane autophagic-like vesicles containing partially undigested material. Features of mitophagy (the presence of mitochondria within autophagosomes), multivesicular bodies and phagophores (a site of autophagosome formation) were also scored (Figure 6C).

### Functional role of autophagy in GVS-treated GBM CSCs

The functional significance of the autophagy induced by (or in response to) GVS treatment was assessed using different known autophagy inhibitors/inducers (Klionsky et al., 2016).

**Figure 7:**
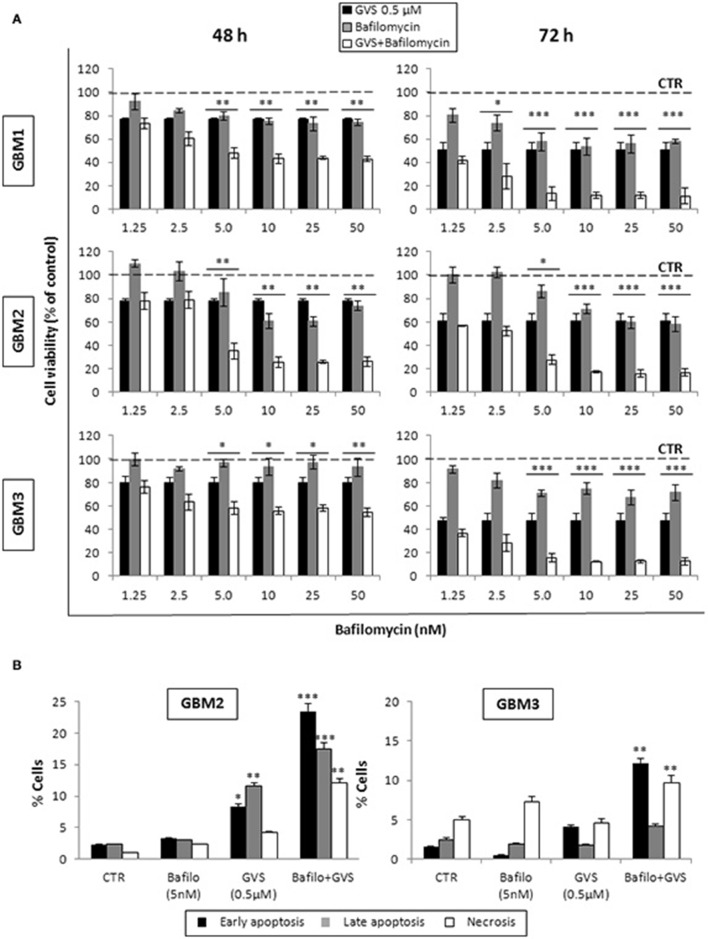
**Evaluation of the role of autophagy in GVS inhibitory effect on GBM CSCs**. **(A)** GBM1, GBM2 and GBM3 CSCs were treated with GVS 0.5 μM (black bars), bafilomycin-A1 1.25, 2.5, 5, 10, 25, and 50 nM (gray bars) or with the combination of all doses of the two drugs (pre-treatment with bafilomycin-A1 for 2 h; white bars) for 48 and 72 h. Cell viability was monitored by MTT assay. Statistical significance was calculated using ANOVA followed by Dunnett's *post-hoc* test; (*p < 0.05, **p < 0.01; ***p < 0.001, vs. control). **(B)** *In vitro* assessment of apoptosis in GBM2 and GBM3 cells treated with bafilomycin-A1 (5 nM) or GVS (0.5 μM), alone or in combination. FACS analysis of Annexin V-PI staining was used to measure apoptosis/necrosis. Histograms represent the percentage of each cell population after 48 h of drug exposure. Statistical analysis was performed using ANOVA followed by Dunnett's *post-hoc* test (*p < 0.05, **p < 0.01, ***p < 0.0001 vs. control).

**Figure 8:**
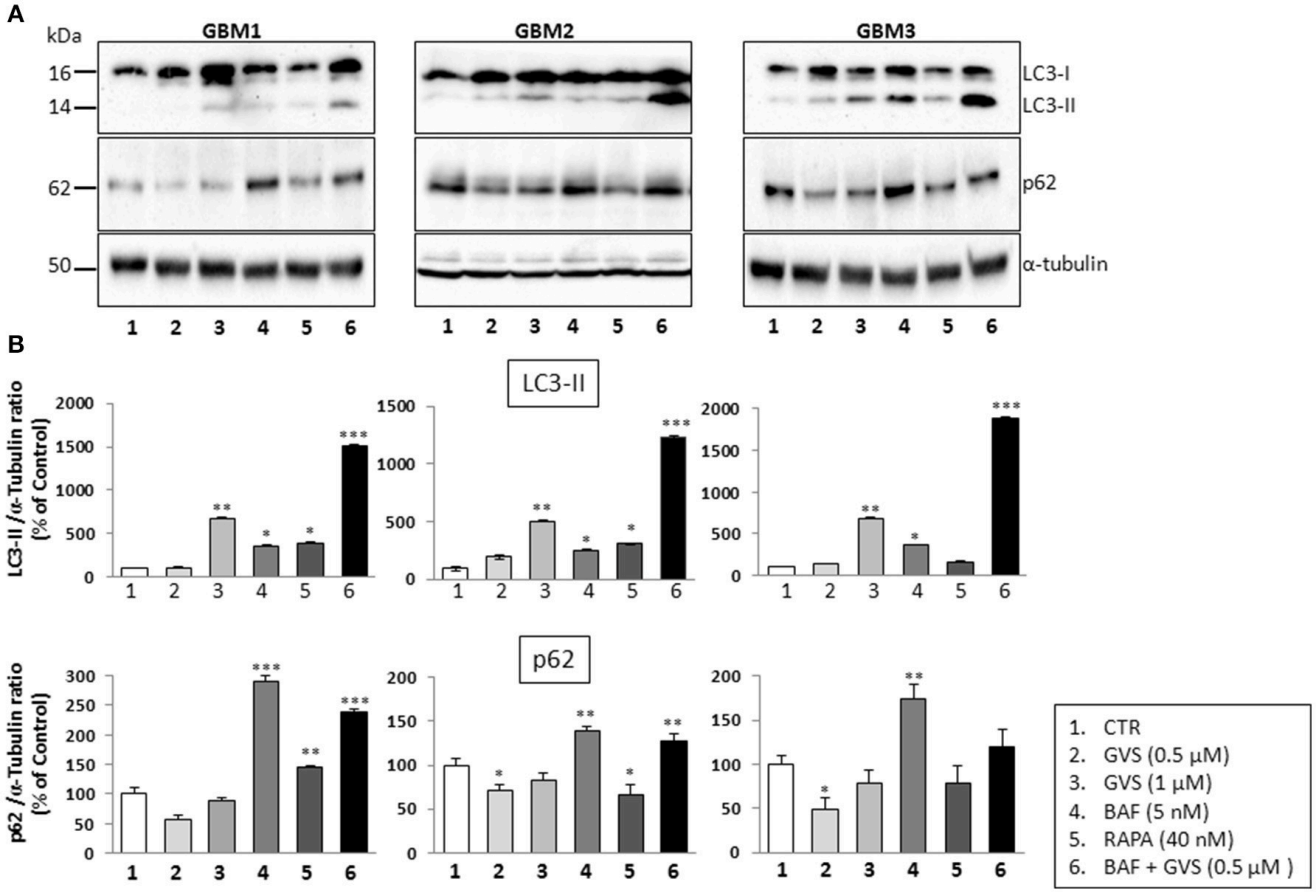
**Effect of the combined treatment with GVS and bafilomycin-A1 on the LC3-II and p62 protein levels**. **(A)** Representative immunoblots of LC3-II and p62 protein levels. Cell lysates were obtained from GBM1, GBM2, and GBM3 CSCs treated with GVS (0.5–1.0 μM), bafilomycin-A1 (5 nM), rapamycin (40 nM), and with GVS 0.5 μM/bafilomycin-A1 combination (2 h pre-treatment with bafilomycin-A1). α-tubulin content was used as loading control. **(B)** Densitometric analysis of LC3-II and p62 protein levels. Data were normalized against α-tubulin and expressed as percentage of untreated control values. Statistical analysis was performed using ANOVA followed by Dunnett's *post-hoc* test (*p < 0.05, **p < 0.01;***p < 0.001 vs. control) comparing GVS, bafilomycin-A1, rapamycin, GVS+ bafilomycin-A1 treatments to the respective untreated (CTR) GBM CCSs.

To arrest the autophagy process, GBM1, GBM2, and GBM3 CSCs were pre-treated with bafilomycin-A1 (up to 50 nM) for 2 h before being incubated with GVS (0.5 μM). Cell viability was evaluated by MTT assay. Forty-eight hours of combined treatment with bafilomycin-A1 and GVS induced a significant decrease in cell viability, compared to bafilomycin-A1 or GVS alone (the combination of bafilomycin-A1+GVS reduced GBM1 viability of 57% compared to −25% of bafilomycin-A1 and −23% GVS; in GBM2 the combined treatment caused a reduction of −74%, in comparison to bafilomycin-A1 −31% and GVS −20%; while in GBM3 we observed −46% viability after bafilomycin-A1+GVS, compared to −8% of bafilomycin-A1 and −20% GVS), with a further increase in the cytotoxic effect induced by the combined treatment after 72 h (Figure 7A). To determine the statistical significance of the GVS/bafilomycin-A1 association, the combination index (CI) values were calculated and plotted using the CompuSyn software (Supplementary Figure 7): CI values above 1 are indicative of antagonism, while below 1 of synergism. GBM1, GBM2, and GBM3 showed CI values <1 starting from the combination GVS 0.5 μM/bafilomycin-A1 2.5 nM for GBM1 (range, 0.2–0.6 at 48 h; 0.07–0.6 at 72 h), GVS 0.5 μM/bafilomycin-A1 5 nM for GBM2 (range, 0.3–0.9 at 48 h, 0.3–0.9 at 72 h) and GVS 0.5 μM/bafilomycin-A1 2.5 nM for GBM3 (range: 0.4–0.6 at 48 h; 0.02–0.4 at 72 h), confirming a synergistic effect exerted by GVS in association with the autophagy inhibitor bafilomycin-A1. Similar results were obtained when the effects of the combined GVS (0.5 μM)/bafilomycin-A1 (5 nM) treatment was investigated on the induction of apoptosis in GBM2 and GBM3 CSCs. As compared to single drug treatments, the drug combination increased the percentage of apoptotic cells (GBM2: 41.27% of early+late apoptotic cells with the association, vs. 19.84 and 6.25% for GVS and bafilomycin-A1, respectively; for GBM3, 16.4% of early+late apoptotic cells with the association, vs. 5.89 and 6.25% for GVS and bafilomycin-A1, respectively). Also the percentage of PI-positive, necrotic cells was increased with the combined treatment with GVS and bafilomycin-A1 (Figure 7B). These results suggested that the inhibition of autophagy by bafilomycin-A1 facilitated GVS-dependent apoptotic cell death in GBM CSCs. Furthermore, GVS+bafilomycin-A1 treatment produced an increase of LC3-II and p62 content, as compared to the amount of these proteins following individual drugs treatments. Conversely, in presence of the autophagy-activator rapamycin or with GVS alone, p62 protein levels were reduced as a result of the increase in autophagy-mediated protein degradation (Figures 8A,B).

**Figure 9:**
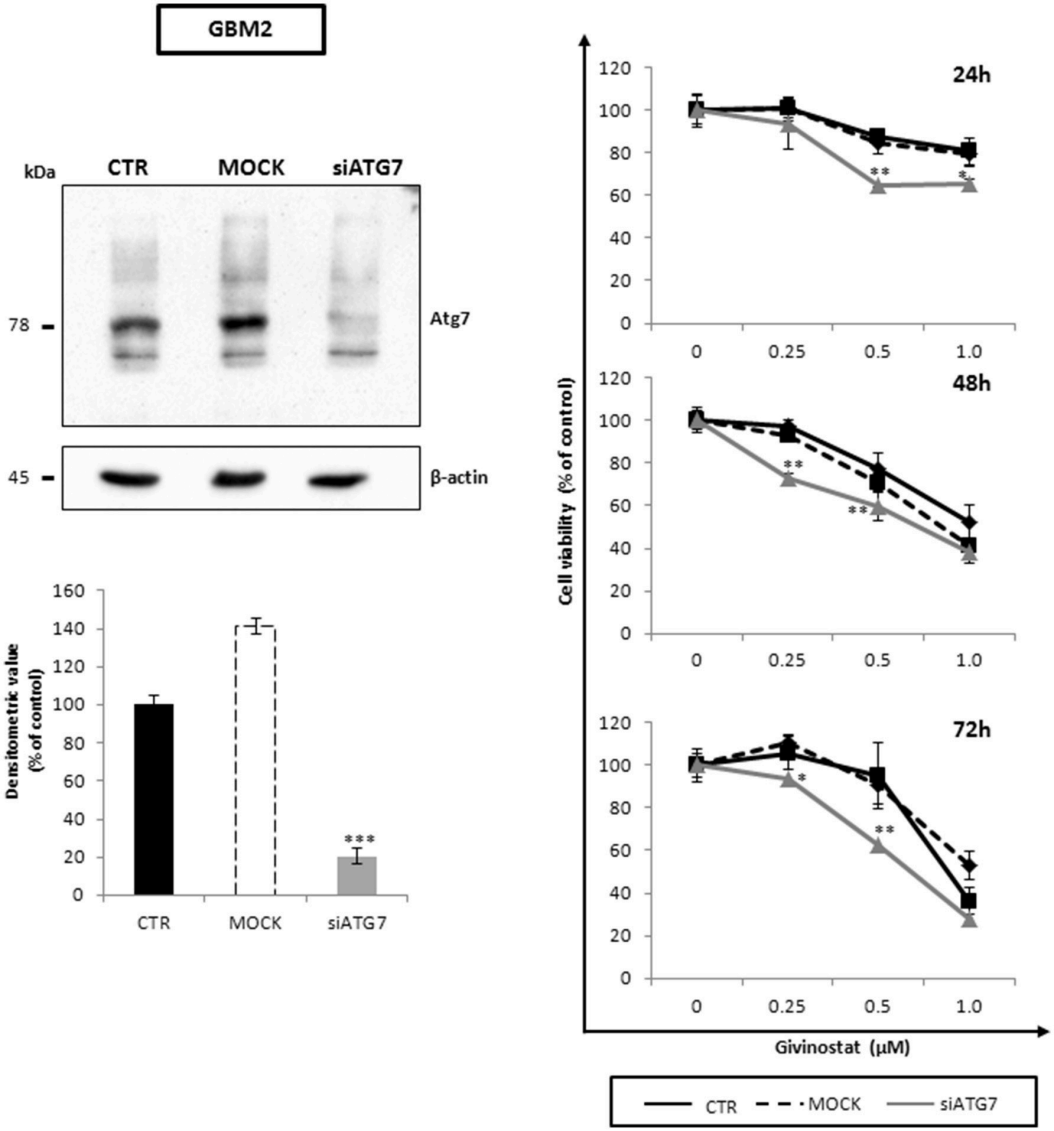
**Atg7 silencing effects on cell viability along with GVS in GBM CSCs**. Equal amount of proteins obtained from GBM2 control (CTR), GBM2 transfected without si*ATG7* (MOCK) and GBM2 transfected with si*ATG7* were assayed. Immunoblotting analysis (left panels) was performed, 24 h post-transfection, in order to ensure *ATG7* silencing and Atg7 downregulation. β-actin was used as loading control. MTT cell viability assay (right panels) was performed at 24, 48, and 72 h after GVS administration (0.25, 0.5, and 1.0 μM), in CTR, MOCK and si*ATG7* transfected GBM2 cells. Statistical analysis was performed using ANOVA followed by Dunnett's *post-hoc* test (*p < 0.05, **p < 0.01; ***p < 0.001 vs. control).

To better decipher the role of autophagy in GVS antitumor activity, GBM2 CSCs were transfected with si*ATG7* validated sequences, to arrest autophagy by reducing the intracellular content of Atg7. The efficacy of gene silencing was verified 24 h post-transfection when Atg7 protein was significantly down-regulated as compared to mock and not-transfected cells (Figure 9, left panel). Treatment of si*ATG7*-GBM2 with GVS caused a further decrease of cell viability (Figure 9, right panel), confirming that the arrest of autophagy enhances GVS tumor cytotoxicity, as also showed by bafilomycin-A1 treatment.

Finally, a widely adopted culture-starvation scheme was applied to activate autophagy in GBM1 and GBM2 CSCs. In detail, CSCs were deprived of growth factors for 60 h before being treated with GVS (up to 2 μM) and cell viability measured by MTT assay. Deprivation of growth factors prevented GVS-dependent cell death, and the activation of autophagy in these conditions was demonstrated by increased LC3-II and reduction in p62 protein levels (Supplementary Figure 8).

## Discussion

In this paper we report that in human GBM stem cells, the inhibition of autophagy using both chemical and RNAi approaches increases the antitumor efficacy of givinostat.

Givinostat (ITF2357, GVS) is a hydroxamic acid-derivative, orally active, pan-HDACi. GVS has anti-inflammatory, anti-neoplastic and anti-angiogenic properties (Leoni et al., 2005), with pro-apoptotic effects in hepatocellular carcinoma (Armeanu et al., 2005), non-small cells lung cancer (Del Bufalo et al., 2014) and acute myeloid leukemia cells (Galimberti et al., 2010).

We report the efficacy of GVS in reducing viability of human GBM CSCs, the subpopulation responsible for GBM resistance to conventional therapy and patients' fatal outcome (Bonavia et al., 2011; Chen et al., 2012; Florio and Barbieri, 2012). *In vitro*, CSCs retain genotypic and phenotypic features of the tumor they are derived from, which are progressively lost when the cells are grown in FBS-containing media, as occurs in established cell lines (Lee et al., 2006). Thus, results obtained using CSC-enriched cultures possess a higher traslational impact than those obtained adopting established cell lines. CSC-enriched cultures, obtained from nine human GBMs, were cultured both as spheroids and monolayers retaining the stem-like biological and phenotypical characteristics (expression of stem cell markers, multilineage differentiation, self-renewal, and tumorigenicity *in vivo*; Griffero et al., 2009; Wurth et al., 2013).

Firstly, we documented that GVS powerfully reduces viability of all CSCs analyzed, independently from the genotypical and phenotypical heterogeneity observed in the GBMs: we observed a prolonged pharmacological activity that could be useful during *in vivo* experimental schemes. GVS also inhibited CSC self-renewal, a mechanism necessary to preserve CSCs within the tumor microenvironment. It is important to highlight that the culture conditions used to grow CSCs (non-adherent tumor-spheres or monolayers in the presence of Matrigel) did not affect GVS efficacy. In contrast, differentiated GBM cells or normal stem cells are modestly affected by GVS-induced cell death. Accordingly, previous studies reported that GVS is cytotoxic for hepatoma cell lines but not for normal human hepatocytes (Armeanu et al., 2005), and, in agreement with our results, that MSCs are resistant to GVS at concentrations higher than 1 μM for at least 72 h (Golay et al., 2007). The reduced activity against non-tumor stem cells while impairing CSC viability, renders GVS an interesting candidate for further clinical evaluations. In addition, we report that GVS had a lower effect on differentiated CSC, further supporting the selectivity of GVS against CSC subpopulation.

It was reported that antitumor activity of GVS depends on the activation of apoptotic pathways (Pathil et al., 2006; Golay et al., 2007; Galimberti et al., 2010). We report that the effect of GVS on GBM CSC viability is associated not only with apoptotic cell death but also with the activation of macroautophagy. Autophagy is a central cell degradation system involved in several physiological and pathophysiological events (Rabinowitz and White, 2010), including cancer (Mathew et al., 2009; Macintosh and Ryan, 2013). A functional link between HDACi and autophagy was demonstrated in cancer cells (Zhang and Zhong, 2014). To this regard, we evaluated the capability of GVS to modify the autophagy process in CSCs, measuring the expression of main ATG proteins. In particular, GVS treatment produced increasing amount of key autophagy executory proteins LC3-II and Beclin1, clearly suggesting that the drug administration induced marked differences within the autophagy process. To further confirm this result, the effect of GVS on autophagy was also assessed by autophagosome analysis. Autophagosomes express LC3-II on the outer membranes, while LC3-I isoform, originated by different post-translational modifications, has a typical cytoplasmic diffuse expression pattern. GVS treatment of GBM CSCs, previously transduced with LC3-GFP baculovirus, induced a significant accumulation of discrete fluorescent LC3-GFP puncta, likely representing autophagosomes. Moreover, increased levels of LC3-GFP in the presence of GVS or compounds interfering with the autophagosome-lysosome fusion (i.e., bafilomycin-A1), was indicative of an increase of the synthesis of autophagy-related membranes (autophagy induction) rather than a reduction in vesicle clearance as for autophagy blockage (Klionsky et al., 2016). Moreover, transmission electron microscopy ultrastructural evaluation highlighted a marked increase in autophagy-like vesicles after GVS exposure. Altogether, these molecular and cellular features indicate that GVS treatment induces the activation of autophagy in GBM CSCs.

As widely discussed (Choi et al., 2013; Macintosh and Ryan, 2013; White, 2015), the contradictory role of autophagy in cancer makes difficult to understand whether this mechanism is *per se* beneficial or inhibitory for the malignant progression. The possible dual functional role of autophagy, i.e., promoting programmed cell death fate or conferring a pro-survival favorable phenotype to the cancer cell, can determine the success or failure of anti-cancer therapies (Palumbo and Comincini, 2013). This opposite behavior represents an adaptive response of the tumor cells to the therapy, and is associated to several tumor-environmental conditions, such as specific or off-targets effects of the drug used, tumor cell type and the state of stem-like or differentiated cells (Galluzzi et al., 2015). To decipher the contribution of the autophagy process to the cytotoxic effects of GVS, autophagy in GBM CSCs was positively or negatively modulated using different experimental approaches. To obtain the autophagy inhibition: the down-regulation of key genes and/or proteins and the blockade of the autophagy flux preventing the fusion of autophagosomes with lysosomes; to activate the autophagy process: rapamycin treatment or growing cells in medium deprived of growth factors.

Bafilomycin-A1, used to inhibit the autophagy flux, is a specific inhibitor of the late phase of autophagy that prevents the acidification of the autophagic vacuoles and inhibits the fusion between autophagosomes and lysosomes (Yamamoto et al., 1998). In parallel, the inhibition of autophagy was achieved by down-regulating *ATG7* gene by siRNA. Silencing *ATG7*, or other key autophagy genes, provides an effective strategy to arrest this pathway blocking the autophagosomes formation and the degradation processes (Saiki et al., 2011; Criollo et al., 2012; Klionsky et al., 2016). In this way, we show that the inhibition of autophagy by both molecular and pharmacological approaches, increases the anti-proliferative and pro-apoptotic effects of GVS on GBM CSCs. This result was confirmed in cells in which autophagy was activated by deprivation of growth factors. The depletion of nutrients is one of the main stimuli triggering autophagy (Mizushima and Klionsky, 2007). When the nutrients' supply is limited, cells activate autophagy to generate a source of metabolic substrates to sustain the energetic requirement for survival (Kang and Avery, 2008). We report that deprivation of growth factors in CSCs and the consequent activation of autophagy results in a protective effect against GVS antitumor activity.

**Figure 10:**
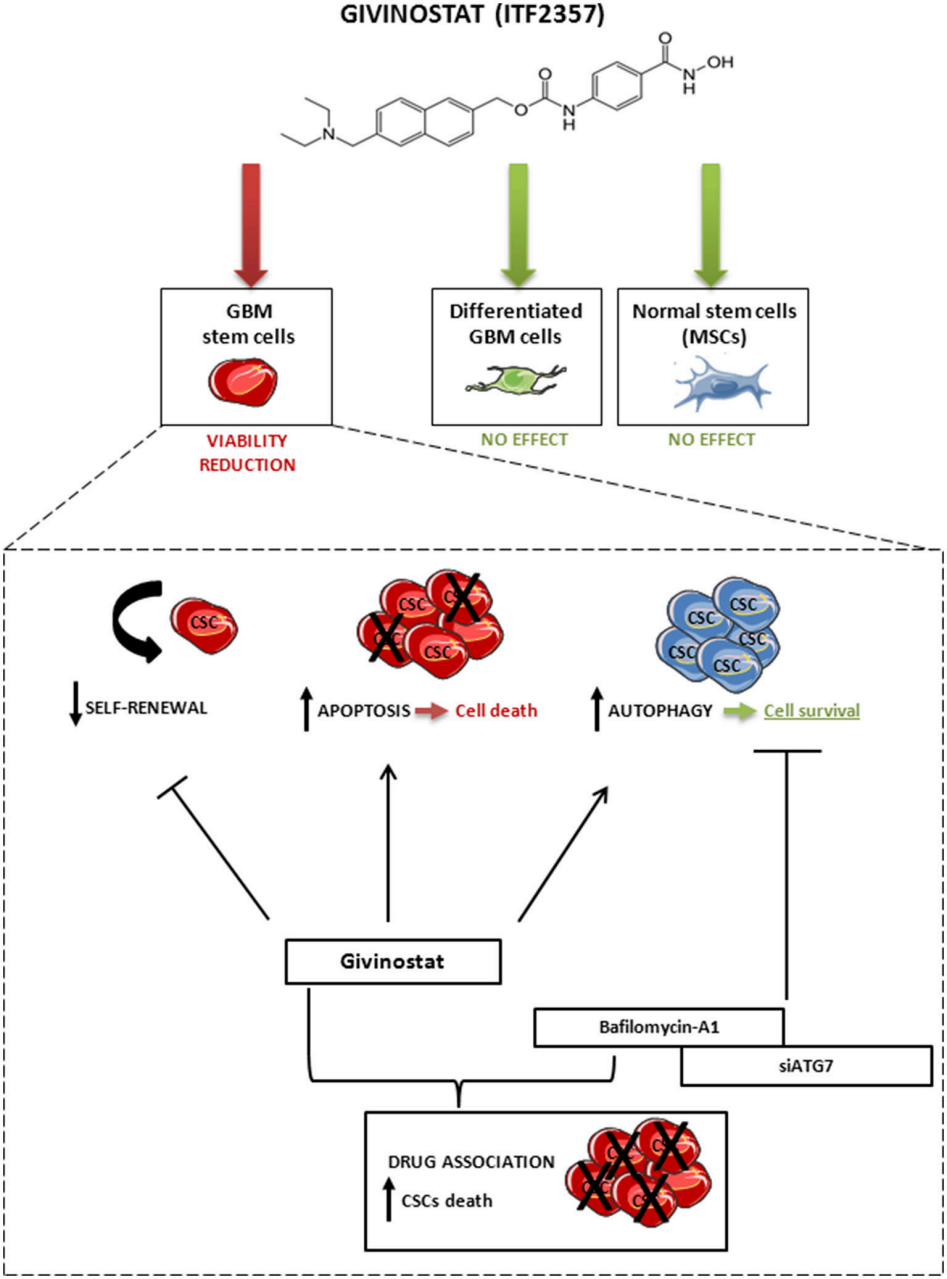
**Schematic representation of the effect of GVS on GBM CSCs, differentiated GBM cells and MSCs**. GVS reduced CSC viability through apoptosis activation and self-renewal impairment; at the same time, GVS induced activation of the autophagy process that resulted in a partial protection of CSCs from GVS pharmacological activity. The autophagy flux arrest, mediated by bafilomycin-A1 co-administration or by *ATG7* silencing, enhanced the GVS pro-apoptotic activity, therefore suggesting a pro-survival effect of the autophagic process to counteract the effect of GVS. Importantly, GVS did not reduce cells viability in differentiated GBM cells and MSCs.

Taken together, these findings suggest that the activation of autophagy occurring during GVS treatment has a cytoprotective effect that, supporting CSC survival, reduces HDACi efficacy. In other terms, tumor cells respond to GVS-induced cell death with the activation of autophagy, a process directed to catabolize damaged proteins and organelles. Lastly, our data indicate that combined treatment with GVS followed by autophagy inhibition has strikingly synergistic anti-tumor activity in GBM, similarly to lung (Del Bufalo et al., 2014), hepatoma (Liu et al., 2010; Yuan et al., 2014), breast (Rao et al., 2012) cancer models. Consistent with these data, we propose a mechanistic evaluation of the possible synergism between GVS effects and autophagy inhibition that enhances the loss of GBM CSC viability (Figure 10).

On the other hand, our results differ from previous studies showing that the combined treatment with imipramine and ticlopidine elicits cell-lethal autophagy in mouse models of gliomagenesis (Shchors et al., 2015). However, it must be pointed out that the quantitatively and qualitatively different modulations of autophagy in our and in the previous study might result in either pro-survival or cytotoxic effects (White et al., 2015).

In conclusion, we demonstrate that GVS is a promising pharmacological agent; its inhibitory activity against CSC proliferation and self-renewal, accomplished with high potency and efficacy, allows us to consider GVS as a novel possible adjuvant approach for GBM treatment. The identification of GVS as a drug specifically directed against CSCs could represent a significant pharmacological alternative for GBM patients. Moreover, our results suggest that, in light of the cellular and biological complexity of GBM, GVS therapeutic efficacy could be intensified by the association with autophagy inhibitors, since these compounds could synergize and revert the resistance to therapeutic interventions of CSCs, and reduce the drug concentration required to achieve a significant tumor mass reduction.

## Author contributions

Conceived and designed the experiments: SC, TF, FB, FA, and GF. Performed the experiments: FA, AP, RW, AD, SC, and AS. Analyzed the data: all the authors. Contributed reagents/materials/analysis tools: SC, TF, and GF. Wrote the manuscript: FA, TF, SC, and GF.

### Conflict of interest statement

GF is an employee of Italfarmaco S.p.A. The other authors declare that the research was conducted in the absence of any commercial or financial relationships that could be construed as a potential conflict of interest.

## Supplementary material

Supplementary Figure 1 **GVS dose-response curves (0.1–2 μM) on U87-MG, U138-MG, and T98G cell viability**. Every 24 h, cell viability was determined by MTT assay for up to 96 h. Experiments were performed in triplicate and the percentage of viability was calculated *vs*. untreated control cell. Statistical analysis was performed with ANOVA test followed by Dunnett's *post-hoc* test (^*^*p* < 0.05, ^**^*p* < 0.01, ^***^*p* < 0.001).

Supplementary Figure 2 **Trypan blue exclusion assay on GBM1 and GBM2 CSCs**. Analysis was performed after GVS (0.5, 1, 2 μM) exposure from 48 to 72 h (black columns represent cells number at T_0_). Data represent the mean and the SD of three independent replicates; (for dead cells: ^*^*p* < 0.05, ^**^*p* < 0.01, ^***^*p* < 0.001, by ANOVA test followed by Dunnett's *post-hoc* test).

Supplementary Figure 3 **Schematic representation of the experimental plan of time-scale cell growth recovery test**.

Supplementary Figure 4 **Cell growth recovery assay performed on GBM1, GBM2, and GBM3 CSCs**. Cells were treated with GVS (0.5 μM), for 24, 48, and 72 h. After each time of exposure, culture medium was replaced with a fresh one devoid of GVS and cell viability was tested by MTT assay at T_0_ (time of medium replacement), and after 24, 48, and 72 h. Histograms indicate the percentage of cell survival compared to untreated control value at T_0_ (^*^*p* < 0.05; ^**^*p* < 0.01, ^***^*p* < 0.001, on ANOVA test followed by Dunnett's *post-hoc* test).

Supplementary Figure 5 **GVS dose-response curves performed on (A) differentiated GBM1 and GBM2 CSCs and (B) human umbilical cord-derived MSCs**. Cell viability was tested after 24–144 h of GVS treatment (0.1–2 μM) and was determined by MTT assay. Experiments were performed in triplicate and percentage of inhibition was calculated vs. untreated control. Statistical analysis was performed with ANOVA test followed by Dunnett's *post-hoc* test (^*^*p* < 0.05, ^**^*p* < 0.01).

Supplementary Figure 6 ***MAP1LC3B* expression in GBM1, GBM2, and GBM3 CSCs**. Cells were treated with GVS (1 μM) for 24, 48, and 72 h and assayed for *MAP1LC3B* mRNA levels by Real time qPCR. Results are given as relative mRNA expression, in arbitrary units of the ratio of the target RNA over *HPRT1* and *TBP* expression levels. Statistical analysis was performed with ANOVA test followed by Dunnett's *post-hoc* test. Bars represent the mean of three independent experiments ± SD (^*^*p* < 0.05; ^**^*p* < 0.01).

Supplementary Figure 7 **CompuSyn software evaluation of the synergistic effect of GVS in combination with bafilomycin-A1 in GBM CSCs**. Isobolograms of drug combination on GBM1, GBM2, and GBM3 CSC viability after treatment for 48 and 72 h, are represented. Combination index (CI) is represented by symbols above (indicate antagonism between drugs) or below the line (indicate synergy) and in the Table on the right.

Supplementary Figure 8 **Effect of deprivation of growth factors on GVS activity in GBM CSCs**. GBM1 and GBM2 CSCs were maintained in the absence of growth factors for 60 h; after this period cells were treated with GVS (0.1, 0.25, 0.5, 1.0, and 2.0 μM) for further 48 h and viability was assessed by MTT assay. In parallel the same study was performed on GBM1 and GBM2 maintained in complete stem medium. Statistical analysis was performed with unpaired two-tailed *t*-test (^*^*p* < 0.05, ^**^*p* < 0.01;^***^*p* < 0.001). To confirm that deprivation of growth factors really increases autophagy, immunoblotting analysis was performed on cell lysates. LC3-I, LC3-II, and p62 protein levels were assayed (right panels).

Supplementary Table 1 **Main clinical-pathological features of tumors, and tumorigenic potential in mice of GBM-derived cell cultures enriched in CSCs**.

Supplementary Table 2 **Inhibition percentage value and statistical significance of GVS antiproliferative effect on GBM CSCs**. Data were obtained from mean percentage of cell viability of treated cells vs. untreated control cells for each concentration and time point of GVS exposure. Statistical analysis was performed with ANOVA test followed by Dunnett's *post-hoc* test; ^*^*p* < 0.05, ^**^*p* < 0.01, ^***^*p* < 0.001 (NS, non-significant; blank boxes, not performed).
