# Supplementary figures and images for: Inhibition of the Autophagy Pathway Synergistically Potentiates the Cytotoxic Activity of Givinostat (ITF2357) on Human Glioblastoma Cancer Stem Cells
Source: Front Mol Neurosci. 2016 Oct 27;9:107. doi: 10.3389/fnmol.2016.00107 (PMC5081386; doi:10.3389/fnmol.2016.00107)

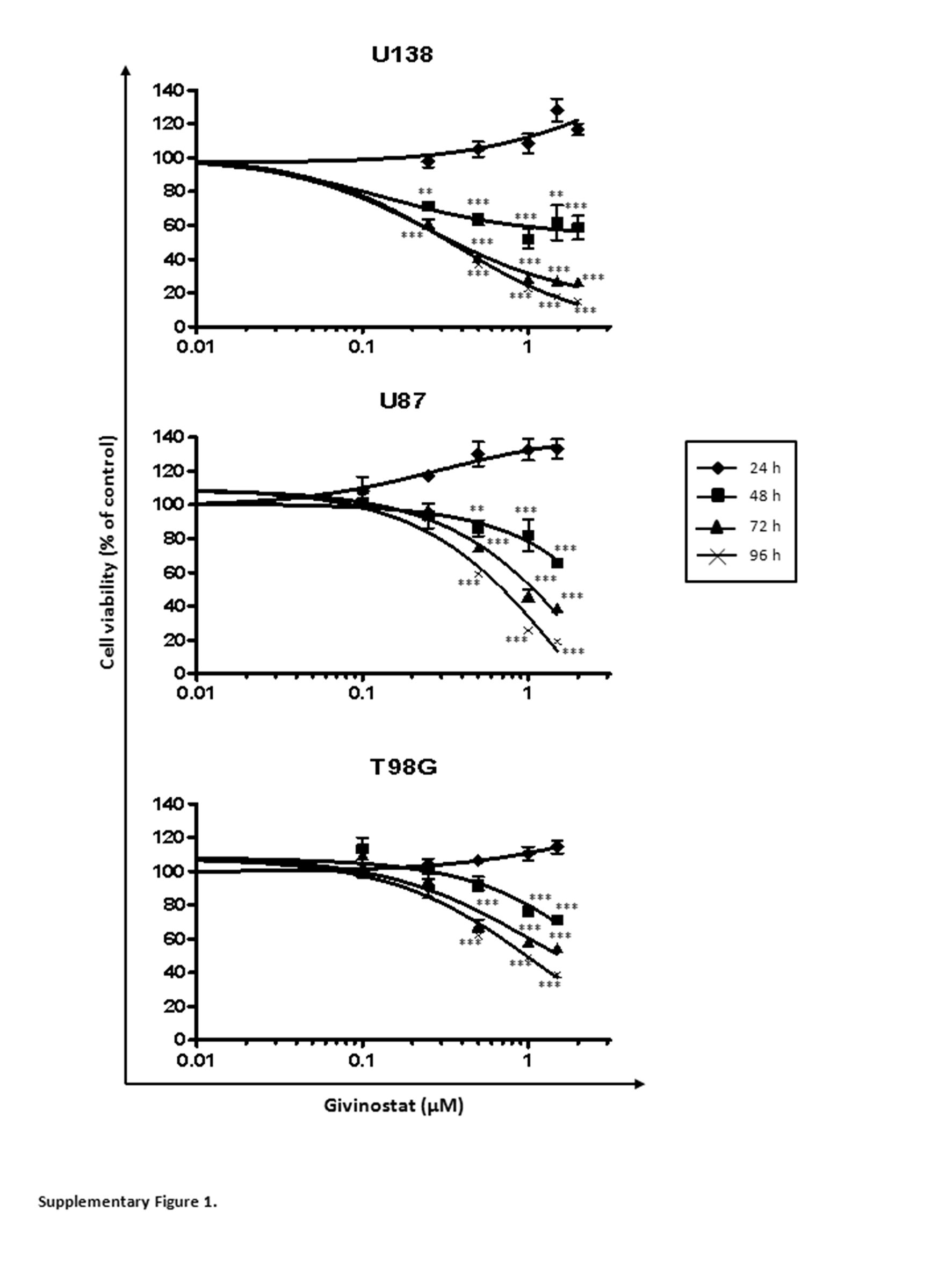

Supplement: Supplementary Figure 1 — GVS dose-response curves (0.1–2 μM) on U87-MG, U138-MG, and T98G cell viability. Every 24 h, cell viability was determined by MTT assay for up to 96 h. Experiments were performed in triplicate and the percentage of viability was calculated vs. untreated control cell. Statistical analysis was performed with ANOVA test followed by Dunnett's post-hoc test (*p < 0.05, **p < 0.01, ***p < 0.001). [file Image1.TIF]

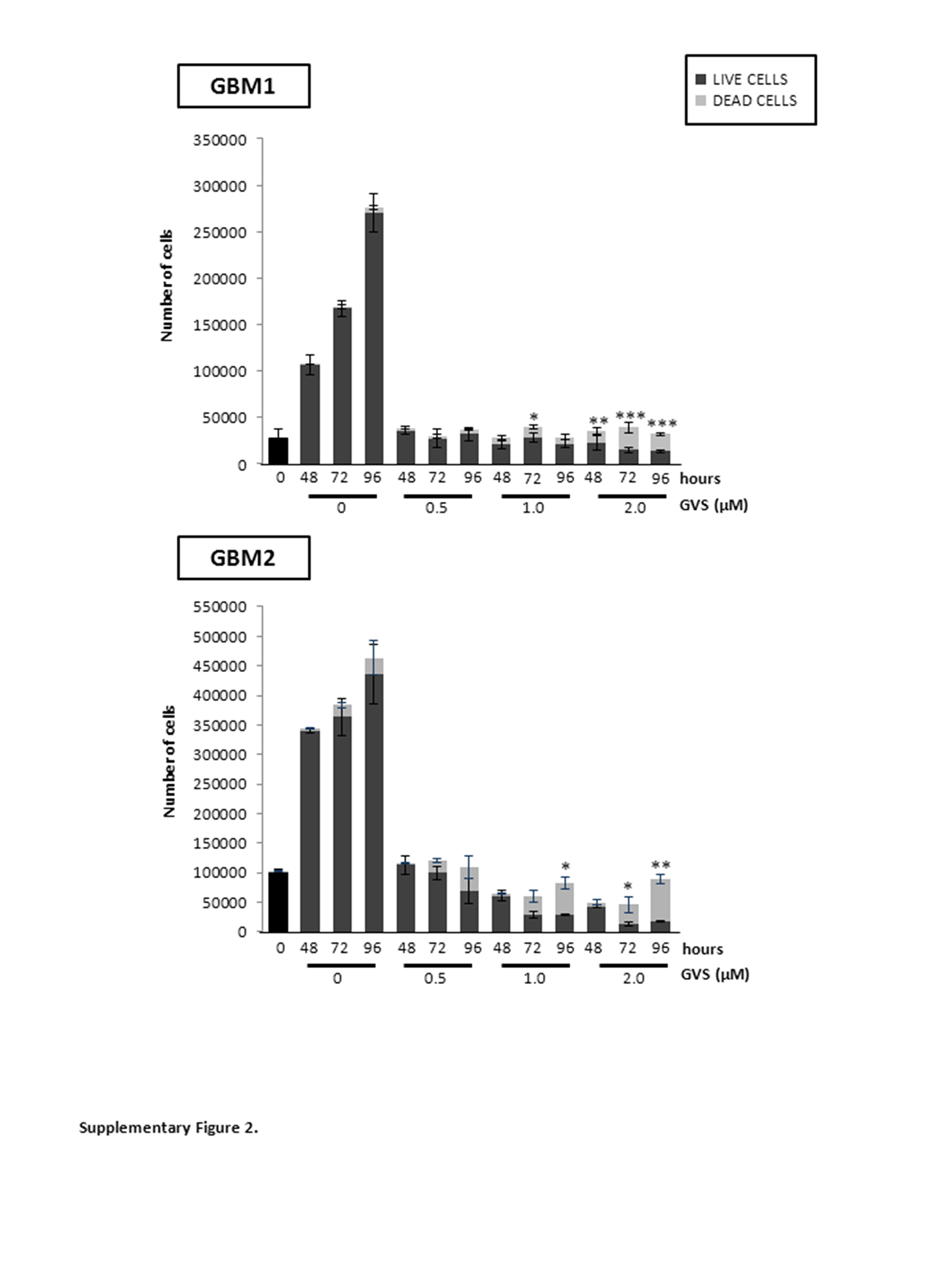

Supplement: Supplementary Figure 2 — Trypan blue exclusion assay on GBM1 and GBM2 CSCs. Analysis was performed after GVS (0.5, 1, 2 μM) exposure from 48 to 72 h (black columns represent cells number at T0). Data represent the mean and the SD of three independent replicates; (for dead cells: *p < 0.05, **p < 0.01, ***p < 0.001, by ANOVA test followed by Dunnett's post-hoc test). [file Image2.TIF]

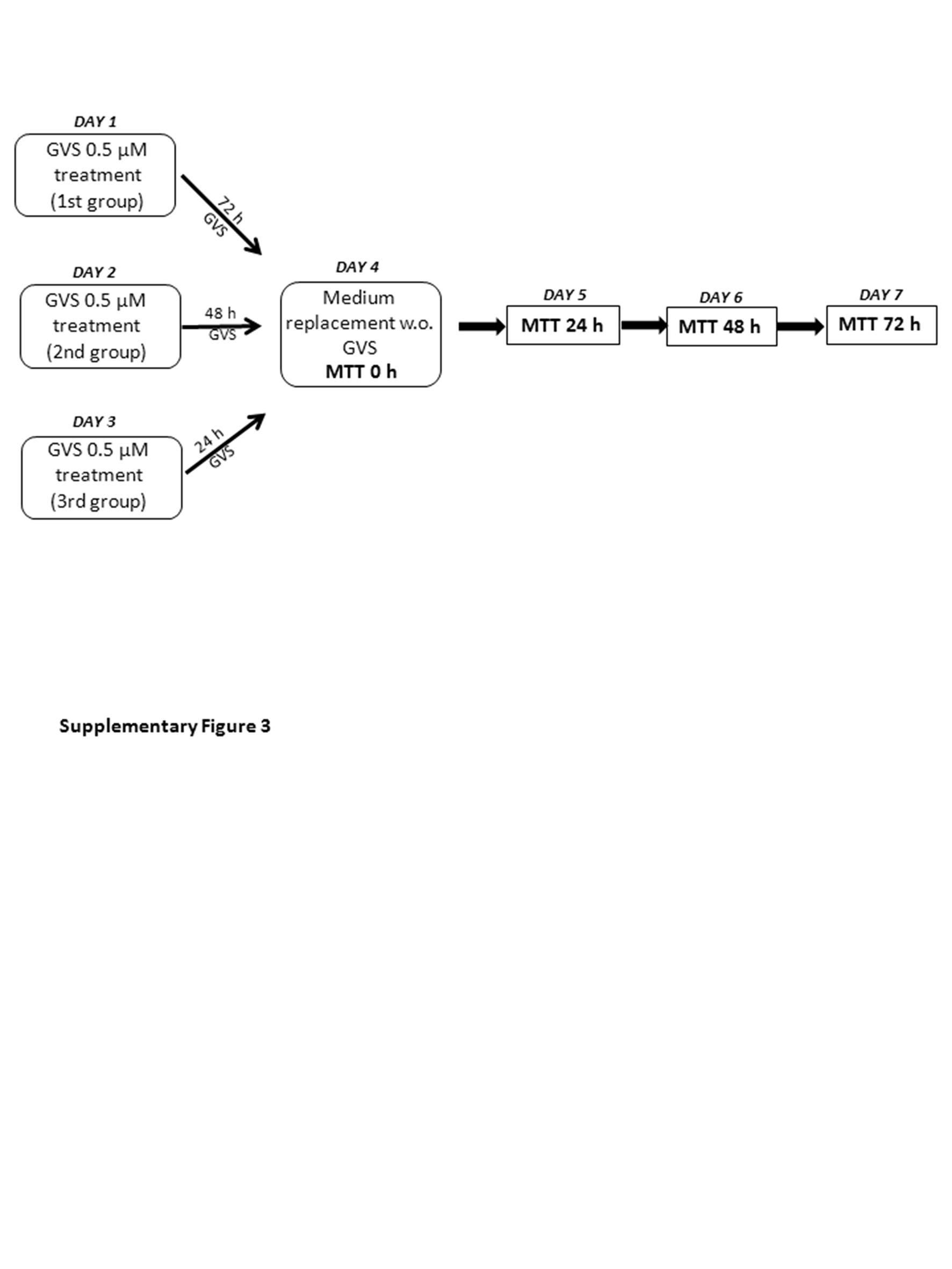

Supplement: Supplementary Figure 3 — Schematic representation of the experimental plan of time-scale cell growth recovery test. [file Image3.TIF]

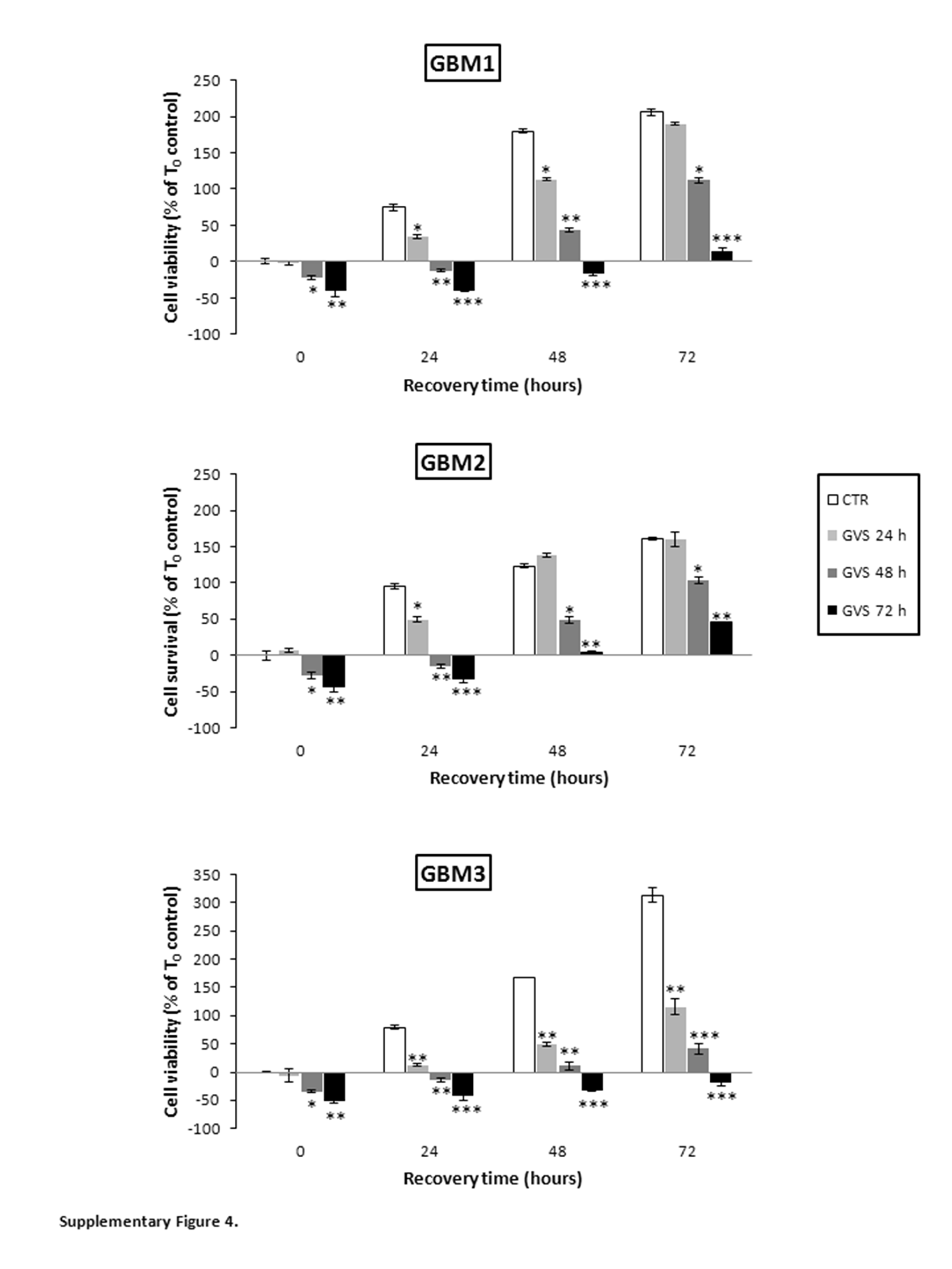

Supplement: Supplementary Figure 4 — Cell growth recovery assay performed on GBM1, GBM2, and GBM3 CSCs. Cells were treated with GVS (0.5 μM), for 24, 48, and 72 h. After each time of exposure, culture medium was replaced with a fresh one devoid of GVS and cell viability was tested by MTT assay at T0 (time of medium replacement), and after 24, 48, and 72 h. Histograms indicate the percentage of cell survival compared to untreated control value at T0 (*p < 0.05; **p < 0.01, ***p < 0.001, on ANOVA test followed by Dunnett's post-hoc test). [file Image4.TIF]

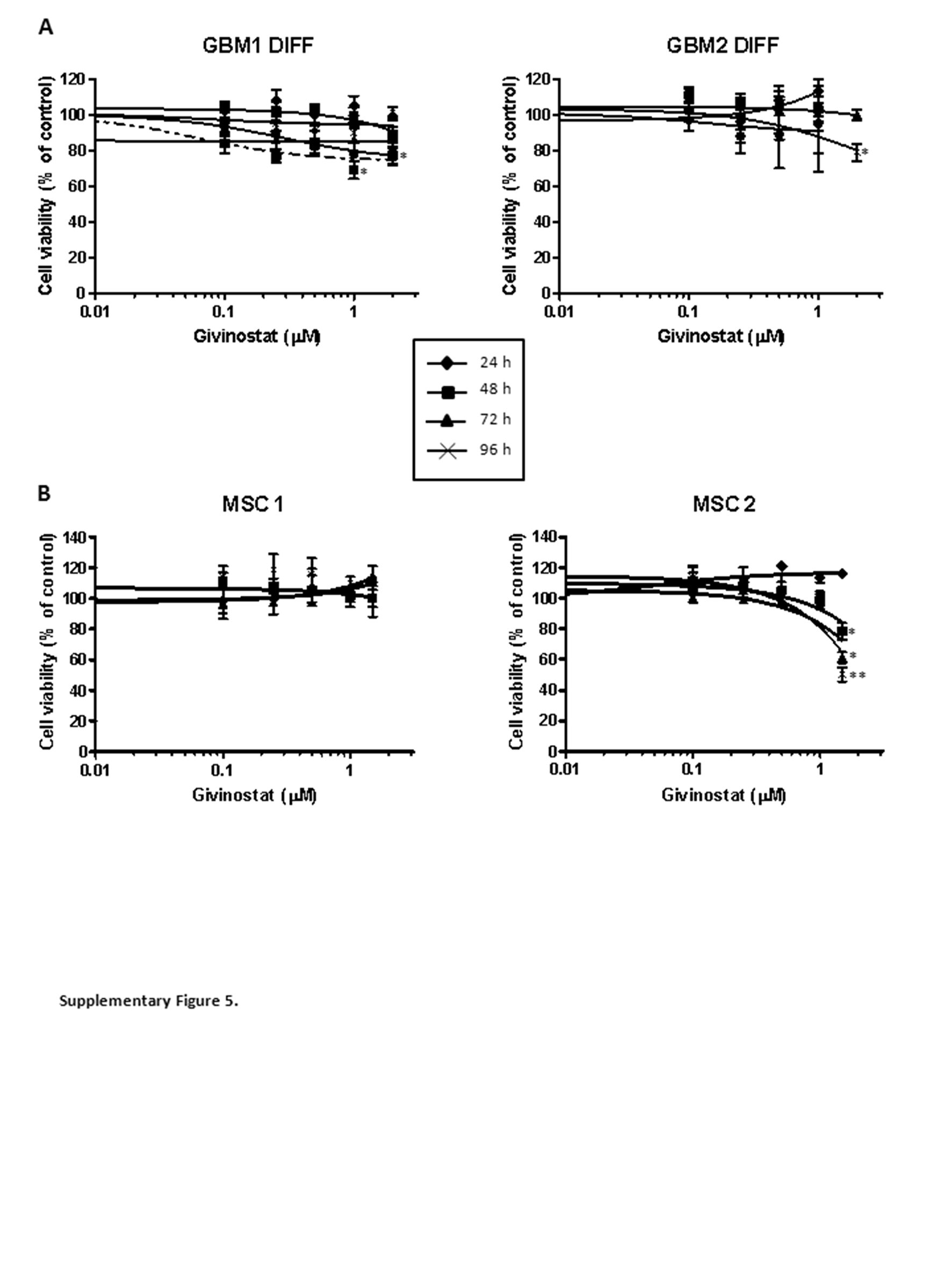

Supplement: Supplementary Figure 5 — GVS dose-response curves performed on (A) differentiated GBM1 and GBM2 CSCs and (B) human umbilical cord-derived MSCs. Cell viability was tested after 24–144 h of GVS treatment (0.1–2 μM) and was determined by MTT assay. Experiments were performed in triplicate and percentage of inhibition was calculated vs. untreated control. Statistical analysis was performed with ANOVA test followed by Dunnett's post-hoc test (*p < 0.05, **p < 0.01). [file Image5.TIF]

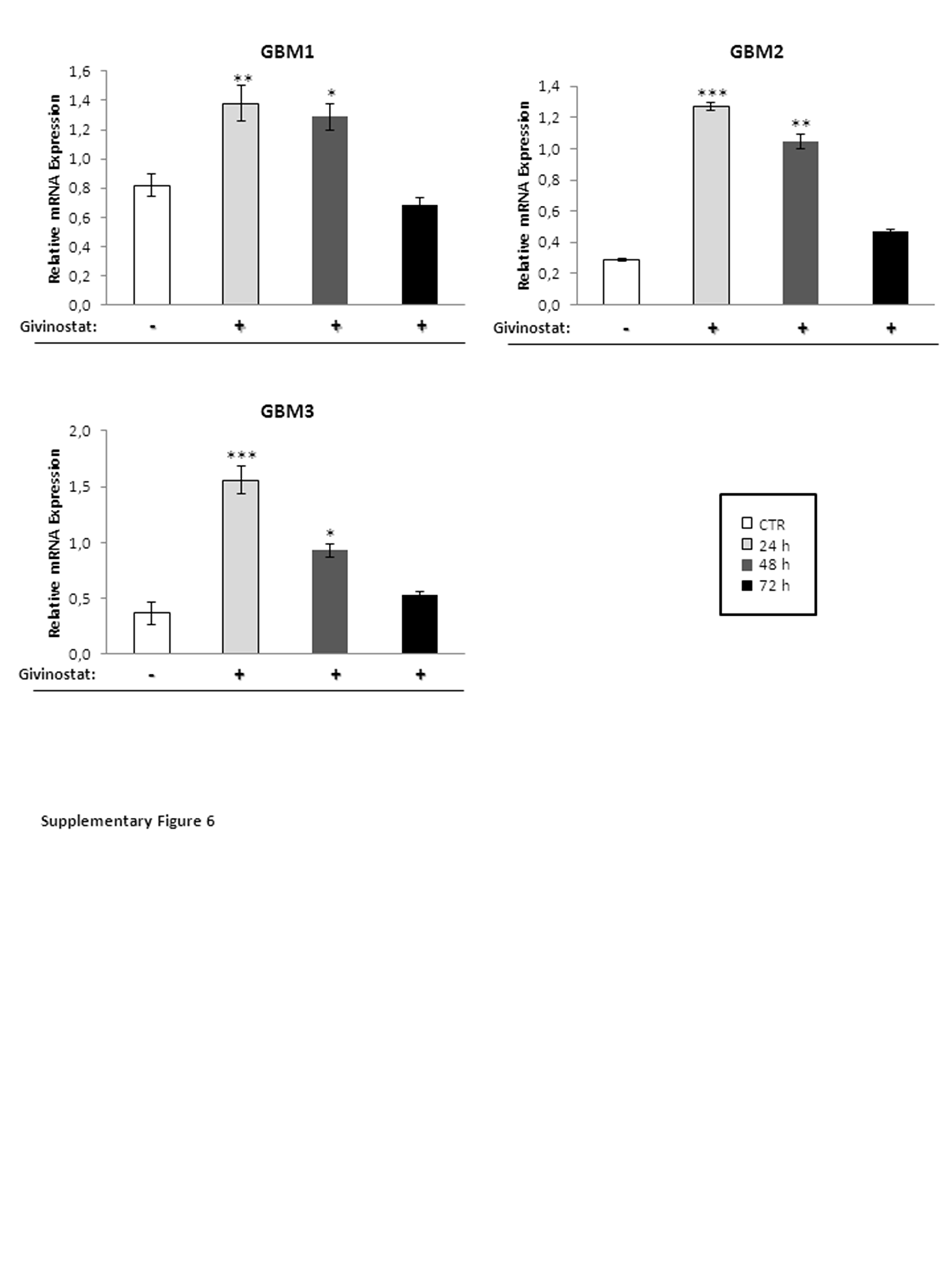

Supplement: Supplementary Figure 6 — MAP1LC3B expression in GBM1, GBM2, and GBM3 CSCs. Cells were treated with GVS (1 μM) for 24, 48, and 72 h and assayed for MAP1LC3B mRNA levels by Real time qPCR. Results are given as relative mRNA expression, in arbitrary units of the ratio of the target RNA over HPRT1 and TBP expression levels. Statistical analysis was performed with ANOVA test followed by Dunnett's post-hoc test. Bars represent the mean of three independent experiments ± SD (*p < 0.05; **p < 0.01). [file Image6.TIF]

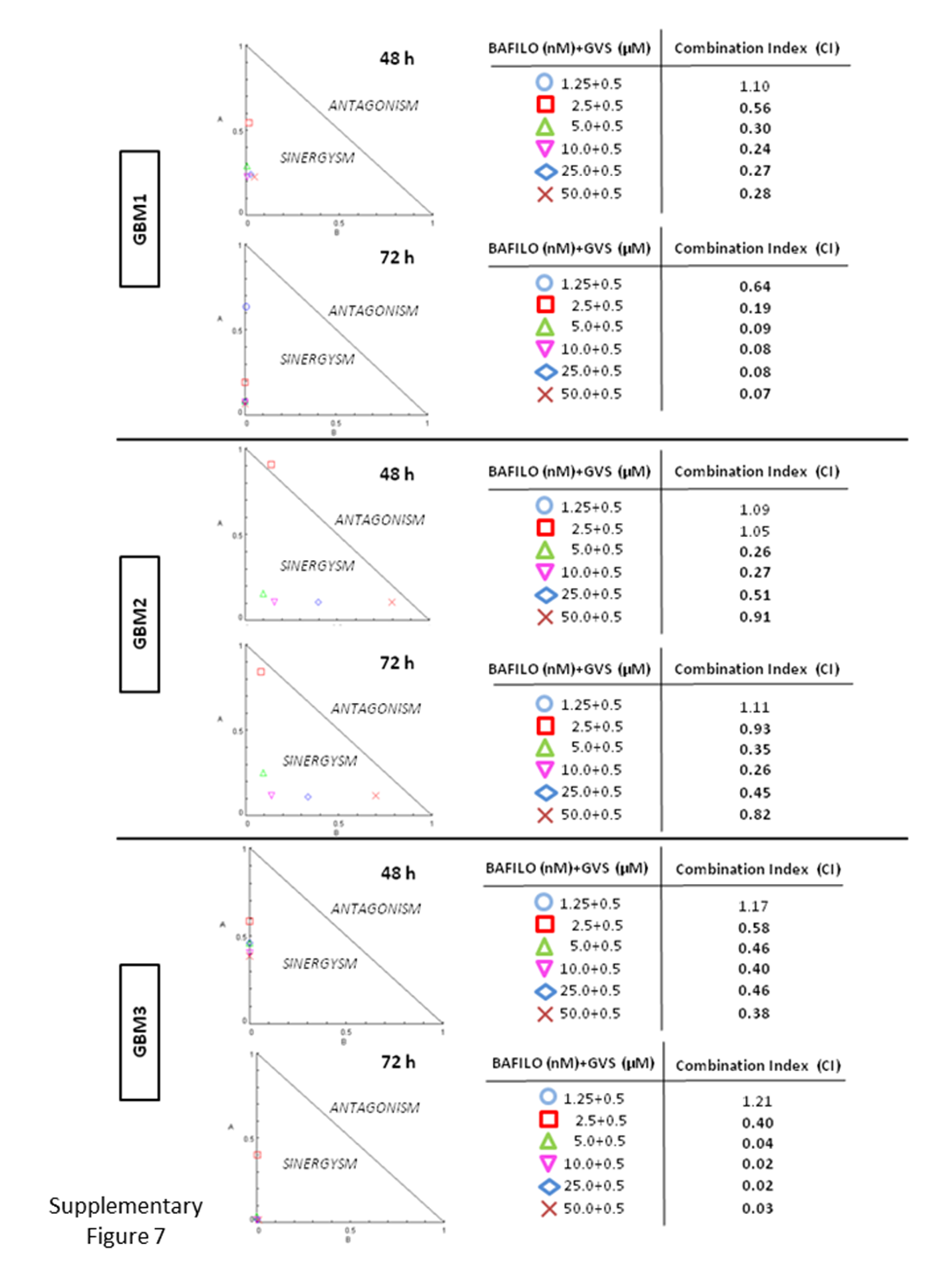

Supplement: Supplementary Figure 7 — CompuSyn software evaluation of the synergistic effect of GVS in combination with bafilomycin-A1 in GBM CSCs. Isobolograms of drug combination on GBM1, GBM2, and GBM3 CSC viability after treatment for 48 and 72 h, are represented. Combination index (CI) is represented by symbols above (indicate antagonism between drugs) or below the line (indicate synergy) and in the Table on the right. [file Image7.TIF]

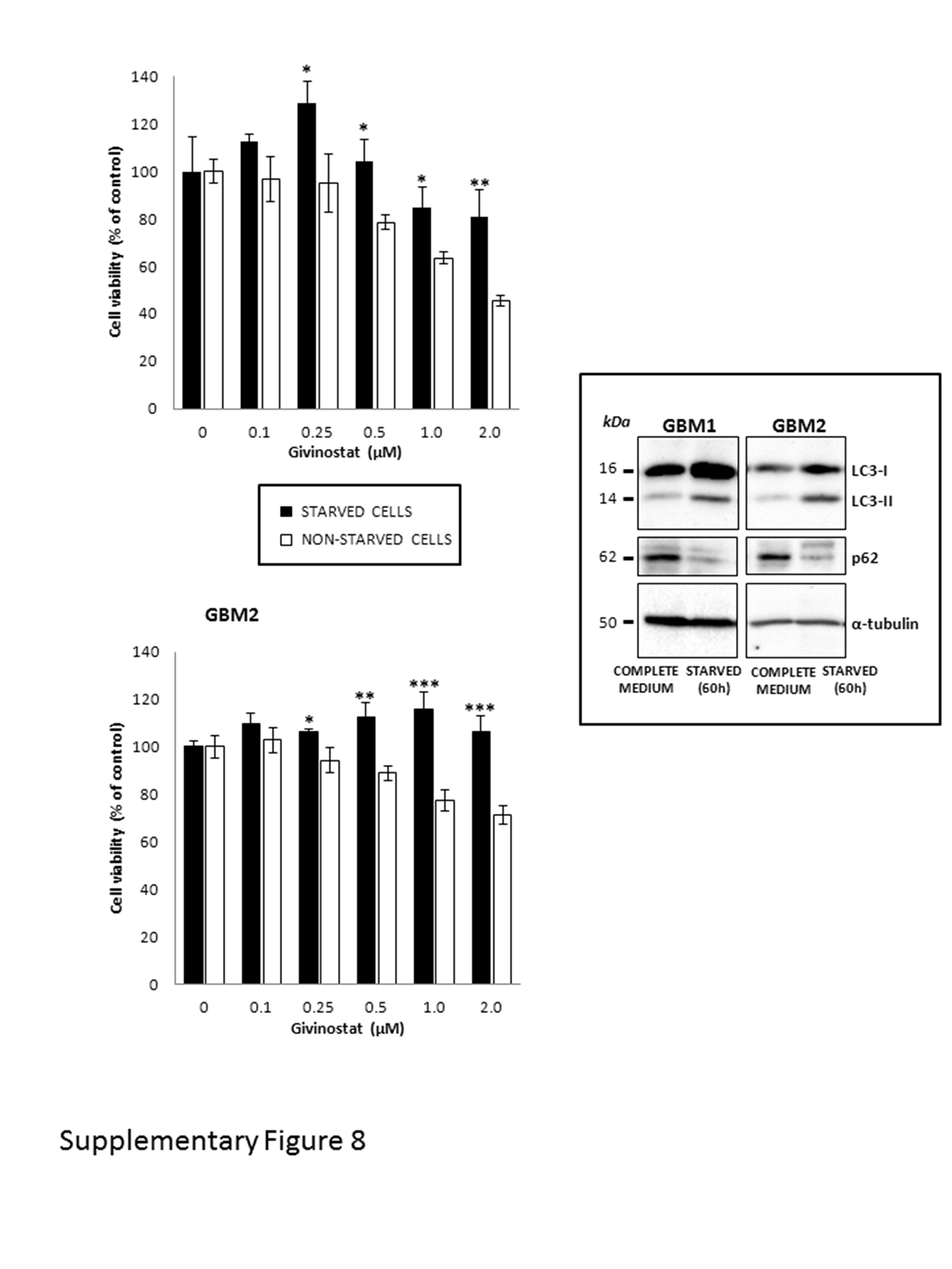

Supplement: Supplementary Figure 8 — Effect of deprivation of growth factors on GVS activity in GBM CSCs. GBM1 and GBM2 CSCs were maintained in the absence of growth factors for 60 h; after this period cells were treated with GVS (0.1, 0.25, 0.5, 1.0, and 2.0 μM) for further 48 h and viability was assessed by MTT assay. In parallel the same study was performed on GBM1 and GBM2 maintained in complete stem medium. Statistical analysis was performed with unpaired two-tailed t-test (*p < 0.05, **p < 0.01;***p < 0.001). To confirm that deprivation of growth factors really increases autophagy, immunoblotting analysis was performed on cell lysates. LC3-I, LC3-II, and p62 protein levels were assayed (right panels). [file Image8.TIF]
