# Supplementary material for: Inhibition of the Autophagy Pathway Synergistically Potentiates the Cytotoxic Activity of Givinostat (ITF2357) on Human Glioblastoma Cancer Stem Cells
Source: Front Mol Neurosci. 2016 Oct 27;9:107. doi: 10.3389/fnmol.2016.00107 (PMC5081386; doi:10.3389/fnmol.2016.00107)
Supplement: Supplementary Table 1 — Main clinical-pathological features of tumors, and tumorigenic potential in mice of GBM-derived cell cultures enriched in CSCs. [file Table1.DOCX]

| **CSCs** | **SEX** | **AGE** | **WHO**  **grade** | **TYPE** | **HEMISPHERE** | **LOBE** | **Ki67** | **Mice survival**  **(*days*)** |
| --- | --- | --- | --- | --- | --- | --- | --- | --- |
| **GBM1** | M | 48 | IV | PRIMARY | LEFT | TEM/PAR/OCC | 40 | 120 |
| **GBM2** | F | 70 | IV | PRIMARY | LEFT | TEM/PAR/OCC | - | 100 |
| **GBM3** | F | 41 | IV | SECONDARY | RIGHT | FR/TEM | 30 | 100 |
| **GBM4** | M | 51 | IV | PRIMARY | RIGHT | TEM/PAR | 40 | 180 |
| **GBM5** | M | 57 | III | SECONDARY | - | - | - | 80 |
| **GBM6** | F | 70 | IV | PRIMARY | RIGHT | FR | 40 | 120 |
| **GBM7** | M | 52 | IV | SECONDARY | LEFT | PAR | 70 | 65 |
| **GBM8** | M | 53 | IV | *RELAPSE* | LEFT | PAR | - | 80 |
| **GBM9** | M | 67 | IV | PRIMARY | RIGHT | TEM/OCC | 50 | 80 |

**Supplementary Table 1.** Main clinical-pathological features of tumors, and tumorigenic potential in mice of GBM-derived cell cultures enriched in CSCs
