# Supplementary material for: Inhibition of the Autophagy Pathway Synergistically Potentiates the Cytotoxic Activity of Givinostat (ITF2357) on Human Glioblastoma Cancer Stem Cells
Source: Front Mol Neurosci. 2016 Oct 27;9:107. doi: 10.3389/fnmol.2016.00107 (PMC5081386; doi:10.3389/fnmol.2016.00107)
Supplement: Supplementary Table 2 — Inhibition percentage value and statistical significance of GVS antiproliferative effect on GBM CSCs. Data were obtained from mean percentage of cell viability of treated cells vs. untreated control cells for each concentration and time point of GVS exposure. Statistical analysis was performed with ANOVA test followed by Dunnett's post-hoc test; *p < 0.05, **p < 0.01, ***p < 0.001 (NS, non-significant; blank boxes, not performed). [file Table2.DOCX]

**Supplementary Table 2.** Inhibition percentage value and statistical significance of GVS on GBM CSCs

|  | **24 h** | | **48 h** | | **72 h** | | **96 h** | | **120 h** | | **144 h** | | **GVS (µM)** |
| --- | --- | --- | --- | --- | --- | --- | --- | --- | --- | --- | --- | --- | --- |
| **GBM1** | +1% | NS | -8% | NS | -18% | NS | -21% | ****** | -38% | ****** | -44% | ****** | 0.1 |
|  | +5% | NS | -6% | NS | -19% | ****** | -32% | ****** | -51% | ******* | -62% | ******* | 0.25 |
|  | +1% | NS | -9% | NS | -31% | ******* | -44% | ******* | -59% | ******* | -71% | ******* | 0.5 |
|  | -3% | NS | -21% | ****** | -40% | ******* | -51% | ******* | -63% | ******* | -77% | ******* | 1.0 |
|  | -3% | NS | -43% | ******* | -53% | ******* | -67% | ******* | -84% | ******* | -87% | ******* | 2.0 |
| **GBM2** | +7% | NS | -15% | NS | -27% | ***** | -28% | ***** | -16% | NS | -43% | ****** | 0.1 |
|  | +3% | NS | -9% | NS | -33% | ****** | -38% | ****** | -51% | ****** | -40% | ****** | 0.25 |
|  | +5% | NS | -16% | ***** | -48% | ******* | -61% | ******* | -76% | ******* | -78% | ******* | 0.5 |
|  | +6% | NS | -25% | ****** | -60% | ******* | -76% | ******* | -85% | ******* | -89% | ******* | 1.0 |
|  | +11% | NS | -43% | ******* | -72% | ******* | -87% | ******* | -90% | ******* | -91% | ******* | 2.0 |
| **GBM3** | +6% | NS | -8% | NS | -15% | NS | -22% | NS | -48% | ****** | -44% | ****** | 0.1 |
|  | +12% | NS | -7% | NS | -28% | ***** | -35% | ***** | -49% | ****** | -76% | ******* | 0.25 |
|  | +9% | NS | -14% | ***** | -39% | ****** | -48% | ****** | -55% | ****** | -80% | ******* | 0.5 |
|  | +7% | NS | -20% | ****** | -44% | ****** | -52% | ****** | -53% | ****** | -84% | ******* | 1.0 |
|  | +12% | NS | -24% | ****** | -66% | ******* | -77% | ******* | -65% | ****** | -87% | ******* | 2.0 |
| **GBM4** | +15% | NS | 0% | NS | -5% | NS | -15% | NS | - | - | - | - | 0.1 |
|  | +10% | NS | -4% | NS | -15% | NS | -20% | ***** | - | - | - | - | 0.25 |
|  | +2% | NS | -13% | NS | -27% | ***** | -34% | ***** | - | - | - | - | 0.5 |
|  | +1% | NS | -17% | ***** | -37% | ****** | -47% | ****** | - | - | - | - | 1.0 |
|  | -4% | NS | -23% | ****** | -40% | ****** | -53% | ****** | - | - | - | - | 2.0 |
| **GBM5** | 0% | NS | +17% | NS | -8% | NS | -12% | NS | -20% | ***** | -30% | ***** | 0.1 |
|  | 0% | NS | +14% | NS | -11% | NS | -28% | ***** | -33% | ***** | -44% | ****** | 0.25 |
|  | -1% | NS | +2% | NS | -21% | ***** | -48% | ****** | -50% | ****** | -62% | ****** | 0.5 |
|  | 0% | NS | +8% | NS | -19% | ***** | -52% | ****** | -65% | ****** | -73% | ******* | 1.0 |
|  | +7% | NS | +1% | NS | -16% | ***** | -53% | ****** | -68% | ******* | -74% | ******* | 2.0 |
| **GBM6** | -2% | NS | +1% | NS | -3% | NS | +1% | NS | -3% | NS | -20% | ***** | 0.1 |
|  | +3% | NS | -2% | NS | -4% | NS | -5% | NS | -6% | NS | -28% | ***** | 0.25 |
|  | +3% | NS | -3% | NS | -7% | NS | -13% | NS | -22% | ***** | -46% | ****** | 0.5 |
|  | +5% | NS | -8% | NS | -21% | ***** | -31% | ****** | -53% | ****** | -70% | ******* | 1.0 |
|  | +6% | NS | -21% | NS | -41% | ****** | -62% | ****** | -82% | ******* | -89% | ******* | 2.0 |
| **GBM7** | +15% | NS | +26% | NS | +6% | NS | +4% | NS | -27% | ***** | -40% | ****** | 0.1 |
|  | +19% | NS | +32% | NS | +10% | NS | -4% | NS | -33% | ***** | -53% | ****** | 0.25 |
|  | +11% | NS | +27% | NS | -6% | NS | -20% | ***** | -40% | ****** | -59% | ****** | 0.5 |
|  | +22% | NS | +16% | NS | -11% | NS | -42% | ****** | -51% | ****** | -68% | ******* | 1.0 |
|  | +30% | NS | +7% | NS | -18% | ***** | -51% | ****** | -60% | ****** | -72% | ******* | 2.0 |
| **GBM8** | +2% | NS | - | - | +8% | NS | -6% | NS | +7% | NS | -1% | NS | 0.1 |
|  | -1% | NS | - | - | +5% | NS | -17% | ***** | -14% | NS | -25% | ***** | 0.25 |
|  | +9% | NS | - | - | -1% | NS | -24% | ***** | -39% | ****** | -52% | ****** | 0.5 |
|  | +6% | NS | - | - | -19% | NS | -43% | ****** | -53% | ****** | -64% | ****** | 1.0 |
|  | +7% | NS | - | - | -33% | ***** | -53% | ****** | -67% | ******* | -75% | ******* | 2.0 |
| **GBM9** | -6% | NS | -2% | NS | -21% | ***** | -15% | NS | -5% | NS | -22% | ***** | 0.1 |
|  | 0% | NS | -7% | NS | -28% | ***** | -25% | ***** | -19% | ***** | -37% | ****** | 0.25 |
|  | +2% | NS | -8% | NS | -34% | ***** | -38% | ****** | -39% | ****** | -56% | ****** | 0.5 |
|  | -3% | NS | -7% | NS | -33% | ***** | -46% | ****** | -60% | ****** | -74% | ******* | 1.0 |
|  | +1% | NS | -19% | ***** | -54% | ****** | -77% | ******* | -84% | ******* | -91% | ******* | 2.0 |

*p<0.05; **p<0.01, ***p<0.001, on ANOVA test followed by Dunnet’s post hoc test. NS, not significant
